# Grazing effects on intraspecific trait variability vary with changing precipitation patterns in Mongolian rangelands

**DOI:** 10.1002/ece3.5895

**Published:** 2019-12-26

**Authors:** Birgit Lang, Julian Ahlborn, Munkhzuul Oyunbileg, Anna Geiger, Henrik von Wehrden, Karsten Wesche, Batlai Oyuntsetseg, Christine Römermann

**Affiliations:** ^1^ Institute of Ecology and Evolution Friedrich Schiller University Jena Germany; ^2^ Faculty of Sustainability Institute of Ecology Leuphana University Lüneburg Lüneburg Germany; ^3^ Botany Department School of Biology and Biotechnology National University of Mongolia Ulaanbaatar Mongolia; ^4^ Senckenberg Museum of Natural History Görlitz Germany; ^5^ International Institute Zittau Technische Universität Dresden Zittau Germany; ^6^ German Centre for Integrative Biodiversity Research (iDiv) Halle‐Jena‐Leipzig Germany

**Keywords:** environmental gradients, grasslands, intraspecific trait variability, land‐use, rainfall, steppes

## Abstract

Functional traits are proxies for plant physiology and performance, which do not only differ between species but also within species. In this work, we hypothesized that (a) with increasing precipitation, the percentage of focal species which significantly respond to changes in grazing intensity increases, while under dry conditions, climate‐induced stress is so high that plant species hardly respond to any changes in grazing intensity and that (b) the magnitude with which species change their trait values in response to grazing, reflected by coefficients of variation (CVs), increases with increasing precipitation. Chosen plant traits were canopy height, plant width, specific leaf area (SLA), chlorophyll fluorescence, performance index, stomatal pore area index (SPI), and individual aboveground biomass of 15 species along a precipitation gradient with different grazing intensities in Mongolian rangelands. We used linear models for each trait to assess whether the percentage of species that respond to grazing changes along the precipitation gradient. To test the second hypothesis, we assessed the magnitude of intraspecific trait variability (ITV) response to grazing, per species, trait, and precipitation level by calculating CVs across the different grazing intensities. ITV was most prominent for SLA and SPI under highest precipitation, confirming our first hypothesis. Accordingly, CVs of canopy height, SPI, and SLA increased with increasing precipitation, partly confirming our second hypothesis. CVs of the species over all traits increased with increasing precipitation only for three species. This study shows that it remains challenging to predict how plant performance will shift under changing environmental conditions based on their traits alone. In this context, the implications for the use of community‐weighted mean trait values are discussed, as not only species abundances change in response to changing environmental conditions, but also values of traits considerably change. Including this aspect in further studies will improve our understanding of processes acting within and among communities.

## Graphical abstract

We focused on the intraspecific changes of plant traits along a precipitation gradient with changing grazing pressure. In detail, we analyzed the intraspecific trait variability of seven traits (canopy height, plant width, specific leaf area, chlorophyll fluorescence, performance index, stomatal pore area index, and individual aboveground biomass) of 15 common steppe species along a precipitation gradient with different grazing intensities at each precipitation level in Mongolian rangelands. Our results highlight that both species and traits show specific or even opposite variability responses to the precipitation gradient under increasing grazing intensity.

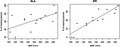

## 1 INTRODUCTION

About 40% of the terrestrial surface is covered by grasslands (Steinfeld et al., 2006; White, Rohweder, & Murray, 2000), which play a crucial role in carbon sequestration, forage production for livestock, and the provision of several other ecosystem services (Rolinski et al., 2015). One important type of grasslands is steppes, especially of temperate Eurasia, ranging from southeastern Europe in the West almost to the Pacific Ocean in the East (Lavrenko, Karamysheva, & Nikulina, 1991; Walter & Breckle, 1994). Next to climate, grazing is one of the main drivers of change in steppe grasslands, affecting morphology and physiology of plants, thereby influencing vegetation composition and the structure and functioning of these ecosystems (Batsaikhan et al., 2014; Wesche et al., 2016; Zheng et al., 2010). Since the late 20th century, animal husbandry and therefore grazing pressure have increased dramatically in several countries due to the privatization of state‐owned cooperatives (Janzen & Bazargur, 2003; Zemmrich, 2006). Land‐use may interact with global climate change, because grasslands are highly influenced by changing climate conditions. In arid systems, moisture availability is the main factor limiting vegetation growth and production (Wesche, 2007). Several studies in rangelands focusing on the influence of different climatic conditions in combination with land‐use intensity predict that grazing responses of plants differ with changing climates (de Bello, Lepš, & Sebastià, 2006; Christensen, Coughenour, Ellis, & Chen, 2004; Díaz et al., 2007; Quiroga, Golluscio, Blanco, & Fernández, 2010; Ruppert et al., 2015). Previous studies of grazing effects have mainly concentrated on the consequences of species turnover in changing community structure and ecosystem function (Ahlborn et al., in revision; Díaz et al., 2004; Grime, 2006; Hooper et al., 2005). Another important factor is the effect of intraspecific trait variability (ITV) of plants, as plant species are typically able to acclimate to changing environmental conditions before they are replaced by other species (Ellenberg, 1996). However, the relevance of ITV has been examined only recently and in most cases from the perspective of community assembly (Jung, Violle, Mondy, Hoffmann, & Muller, 2010; Siefert et al., 2015). Several studies deal with species‐specific trait changes, reflected in both phenotypic responses of individual plants and genotypic differentiation determined by grazing (Mason, Bello, Doležal, & Lepš, 2011; Münzbergová, Hadincová, Skálová, & Vandvik, 2017; Völler, Bossdorf, Prati, & Auge, 2017).

Grassland plants often reduce size under regular grazing (Díaz et al., 2007), increase biomass allocation to vegetative organs (Niu, Choler, Zhao, & Du, 2009), increase belowground growth (López‐Mársico, Altesor, Oyarzabal, Baldassini, & Paruelo, 2015; Oesterheld, 1992), and enhance foliar nutrient accumulation (Bai et al., 2012). These patterns of ITV caused by different grazing intensities are typically found before species turnover within the plant community takes place (Volf et al., 2016). This is particularly relevant for grasslands with mainly perennial species where grazing can have relatively small effects on species relative abundance or occurrence in the short term (de Bello, Lepš, & Sebastià, 2007; Cingolani, Posse, & Collantes, 2005; Volf et al., 2016). Previous studies have typically assumed that ITV is small compared to interspecific variation (Kraft, Cornwell, Webb, & Ackerly, 2007), while a number of recent publications have emphasized that ITV is important for plant community assembly and ecosystem functioning (Siefert et al., 2015; Violle et al., 2012). Due to species responses to environmental changes, ITV may also contribute strongly to changes in community mean trait values (Jung et al., 2010; Lepš, Bello, Šmilauer, & Doležal, 2011). Patterns of species responses to environmental changes and their impact on their environment in terms of changing ecosystem services are supposedly influenced by ITV (Albert et al., 2010), but the magnitude of the effect is mostly unclear because in most studies species are described by mean trait values without any consideration of ITV. However, few studies revealed the influence of abiotic environmental factors on grazing‐induced ITV. Zheng et al. (2011), for instance, analyzed trait responses of two steppe species in response to grazing in 2 years with different precipitation, and they found that the magnitude of trait change for each species was greater in a wet year than in a dry year.

Until now, only a few of the recent studies in the Mongolian rangelands quantified the combined effects of climate and land‐use on functional traits of single species (e.g., Liu et al., 2010; Zheng et al., 2011). Thus, more knowledge is needed to understand the joint effects of changes in climate and land‐use on plant species reactions in this region. Different studies already showed that changes in biomass production are driven by changes in moisture availability (Fernandez‐Gimenez & Allen‐Díaz, 1999; Miehe, Kluge, Wehrden, & Retzer, 2010). However, only a handful multi‐site analyses exist in grasslands in general that include the interacting effect of grazing intensity and moisture availability (Ahlborn et al., in revision; de Bello et al., 2006; Wang et al., 2017). Studies covering both precipitation and grazing gradients are necessary to understand and eventually predict species‐specific responses to grazing under changing climatic conditions for the next decades.

In this study, we examined the effects of grazing on intraspecific variability of plant functional traits in Mongolian rangelands. Mongolia hosts the largest intact part of the Eurasian steppe biome, and possibly one of the most impressive grasslands globally (Batsaikhan et al., 2014). We investigated five grazing intensities at fourteen sites along a precipitation gradient. We selected 15 focal species and measured functional traits related to plant growth such as canopy height, plant width, and biomass, which are highly influenced by grazing and moisture availability (Table 1; An & Li, 2014; Díaz et al., 2007). As a measure for plant performance in terms of growth rate and photosynthesis, we investigated specific leaf area (SLA), chlorophyll fluorescence (*F*_v_/*F*_m_, PI_abs_), and stomatal parameters (Table 1). SLA is related not only to the growth rate, but also to the competitive strength of plants (An & Li, 2014; Zheng, Ren, Li, & Lan, 2012). *F*_v_/*F*_m_ and PI_abs_ serve as a proxy for photosynthesis rate, species fitness (Bucher, Bernhardt‐Römermann, & Römermann, 2018), and water use efficiency (Sinclair, Zwieniecki, & Holbrook, 2008) and are thought to be negatively affected by abiotic and biotic stresses (Maxwell & Johnson, 2000; Römermann, Bucher, Hahn, & Bernhardt‐Römermann, 2016; Zhao, Chen, Han, & Lin, 2009).

**Table 1:** Overview of functional traits measured and analyzed in this study as well as their ecological significance

Trait	Abbreviation	Unit	Ecological significance
Canopy height	—	cm	Proxy for plant growth, competitive vigor (Pérez‐Harguindeguy et al., 2013)
Plant width	—	cm	Proxy for plant growth, competitive vigor (Pérez‐Harguindeguy et al., 2013)
Aboveground biomass	—	g	Proxy for competitive ability, fecundity (Weiher et al., 1999)
Specific leaf area	SLA	mm^2^/mg	Proxy for growth rate (Garnier & Shipley, 2001; Pérez‐Harguindeguy et al., 2013)
Chlorophyll fluorescence	*F*_v_/*F*_m_	nondimensional	Proxy for photosynthesis, plant fitness (Maxwell & Johnson, 2000)
Performance index	PI_abs_	nondimensional	Proxy for sample vitality (Maxwell & Johnson, 2000)
Stomata size	—	μm	Proxy for photosynthesis (Woodward, Lake, & Quick, 2002)
Stomata density	—	number/ mm^2^	Proxy for photosynthesis (Woodward et al., 2002)
Stomatal pore area index	SPI	nondimensional	Proxy for leaf hydraulic conductance and photosynthesis (Sack et al., 2003)

Specifically, this study analyses whether precipitation and grazing intensity have a combined effect on the response of plant species with respect to their trait values. First, we hypothesize that (a) with increasing precipitation the percentage of focal species which significantly respond to changes in grazing intensity increases; while under dry conditions, climate‐induced stress is so high that plant species hardly respond to any changes in grazing intensity. We tested this hypothesis by analyzing the proportion of species responding to changes in grazing intensity in canopy height, plant width, aboveground biomass, SLA, *F*_v_/*F*_m_, PI_abs_, and stomatal pore area index (SPI) along the precipitation gradient. Second, we hypothesize that (b) the magnitude with which species change their trait values in response to grazing, reflected by coefficients of variation (CVs), increases with increasing precipitation. We tested this hypothesis by analyzing the CVs per trait, species, and precipitation level.

## 2 MATERIAL AND METHODS

### 2.1 Study sites

The Mongolian steppes are part of the dry and cold eastern Eurasian grassland covering more than 10 million km^2^ and are characterized by a continental climate with low mean annual precipitation (MAP) ranging from 100 to 300 mm (Wesche et al., 2016). The traditional land‐use system in Mongolia is nomadic pastoralism, which can be seen as a strategy to buffer temporal variability in climate and forage availability by utilizing spatial heterogeneity and moving to less affected regions (Wesche & Treiber, 2012). Palynological evidence implies that many steppe sites have not changed much in the last millennia (Herzschuh, Tarasov, Wünnemann, & Hartmann, 2004).

We chose Mongolia as our model system for three main reasons: First, Central Asia is a dryland region with strong abiotic controls on both plant distribution and performance. Potential effects of differing climate can thus easily be detected. Second, moisture availability is the main controlling factor and a pronounced precipitation gradient facilitates conducting a gradient study with a reasonable effort (Wesche & Treiber, 2012). Last, grazing is the single most important form of land‐use (Zheng et al., 2010). Most of the precipitation (typically more than two‐thirds of the annual mean) falls in the relatively short growing season. This is also the warmest quarter of the year (June to August). Winter precipitation is very low, and usually, springs are also dry (Wesche & Treiber, 2012). Going from the northern to the southern part of Mongolia, aridity increases together with a steady decrease of MAP. In Mongolia, the zoning of the vegetation corresponds rather well with the gradient of decreasing precipitation from the North to the South (van Staalduinen, 2005). Improvements during the last century in both well digging and veterinarian maintenance have led to increased livestock numbers (Fernández‐Giménez et al., 2017). Almost 1.3 million km^2^ of the Mongolian grasslands are intensively grazed rangelands (Sneath, 1998; Wesche & Treiber, 2012). Nevertheless, Mongolian grasslands are still one of the most intact grazing systems in the world (Batsaikhan et al., 2014). Therefore, the steppes of Mongolia served as a perfect test bed for our study.

**Figure 1:**
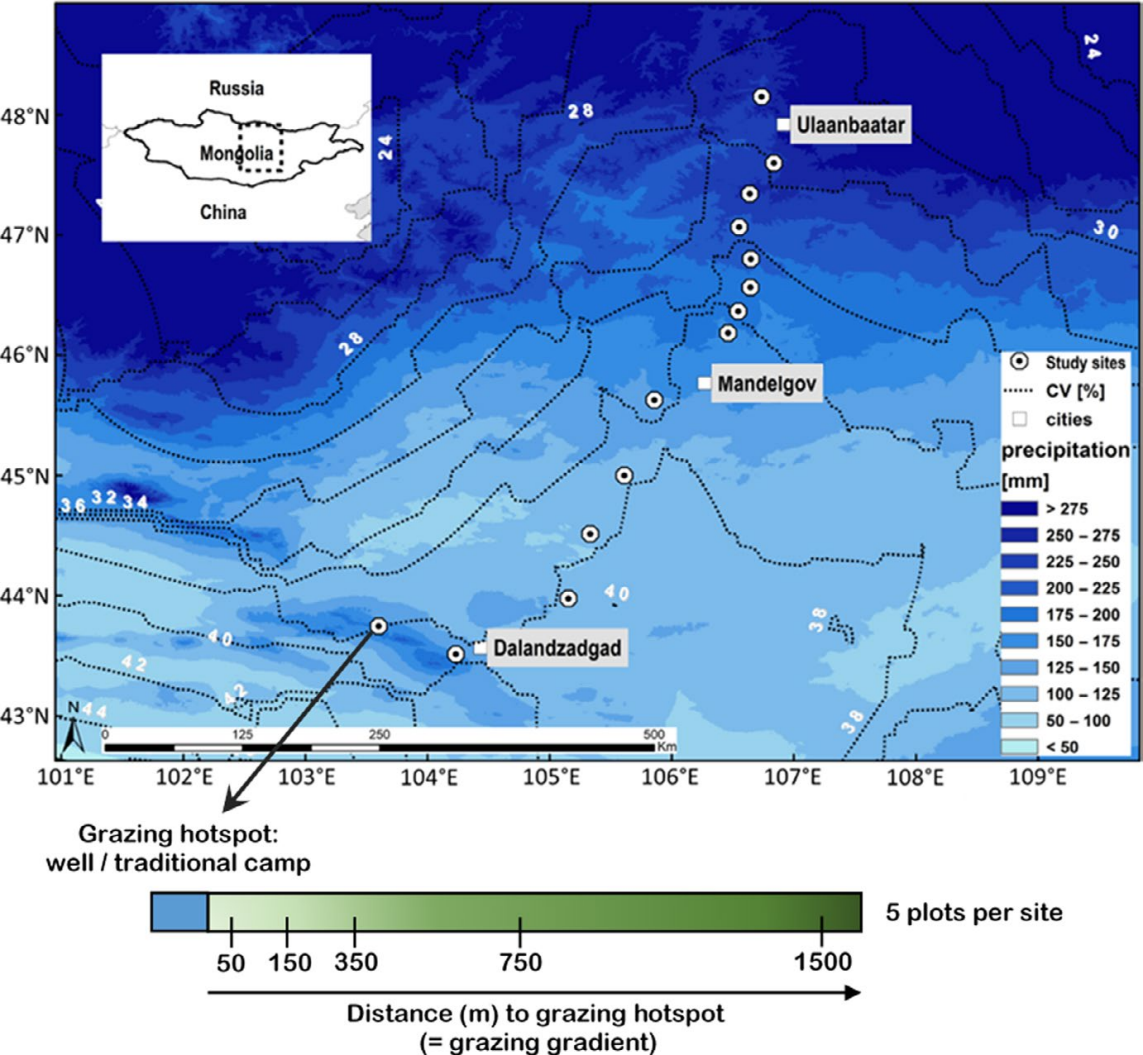
Map of the 14 study sites according to the mean annual precipitation and the coefficient of interannual rainfall variance (CV) in the Mongolian steppe as well as illustration of the grazing gradient per site. The grazing gradient is represented by five plots in different distances (50, 150, 350, 750, 1,500 m) to a grazing hotspot (=well or traditional camp). Figure from Lang et al. (2019), modified. Rainfall data derived from Hijmans et al. (2005), values for coefficient of interannual precipitation variation from Wehrden et al. (2012)

Fourteen study sites were selected along a 600 km precipitation gradient in the steppes of Mongolia ranging from 105 to 250 mm MAP (Figure 1; Hijmans, Cameron, Parra, Jones, & Jarvis, 2005). At each site, we perpendicularly ran land‐use intensity transects, starting from traditionally intensively grazed areas to areas with low intensity of grazing. Five plots (A, B, C, D, and E) were established per site. Plot A was always close (50 m) to grazing hotspots such as water sources or a nomadic camp and therefore represented the highest grazing intensity. Plot B had a distance of 150 m, plot C 350 m, plot D 750 m, and plot E 1,500 m to the nomadic camp or to the water source. Choice of distances was based on previous successful studies in the region (Stumpp, Wesche, Retzer, & Miehe, 2005). In total, we established 70 plots (14 sites × 5 plots).

### 2.2 Species and individual selection

Fifteen dominant species (Table 2) of the Mongolian steppe vegetation with high frequency and individual number were chosen for plant functional trait measurements. The chosen species represent the main biomass producers along the precipitation gradient. The selection comprised mainly herbaceous perennial plants, because all main forage plants utilized by livestock in Mongolia are perennials (Jigjidsuren & Johnson, 2003). Species selection was based on pre‐analyses of existing vegetation data (Hilbig, 1995; von Wehrden, Wesche, & Miehe, 2009). Among these 15 species, three species had their main distribution in the southern desert steppe, six occurred over the whole gradient, and six species had highest abundances in the northern meadow steppe ranging up to the forest steppe belt. Measurements were carried out on eight healthy adult individuals of each species per plot to cover ITV within this plot. All trait measurements were always taken on the same set of individuals within one vegetation period. Not all target species were found at each site (Table S1). Overall, we measured 4,297 individuals.

**Table 2:** Selected species for functional trait measurements along the precipitation and grazing gradient, listed with their main distribution in Mongolia according to Jigjidsuren and Johnson (2003) and Grubov (2001)

Species	Family	Growth form	Habitat/stepptype
*Agropyron cristatum* (L.) Beauv.	Poaceae	Perennial grass	Steppes, steppe soddy, debris, and stony slopes
*Allium polyrrhizum* Turcz. ex Regel.	Alliaceae	Perennial herb	Desert steppes and deserts
*Artemisia adamsii* Bess.	Asteraceae	Perennial herb	Steppe stony mountain slopes and tailings, around springs and lakes, river banks
*Artemisia frigida* Willd.	Asteraceae	Perennial herb	Steppe stony and debris slopes, steppes, and deserts
*Chenopodium album* L.	Chenopodiaceae	Annual herb	Side of alkaline water bodies, often as weed in inhabited areas, nomad camp sites
*Cleistogenes squarrosa* (Trin.) Keng	Poaceae	Perennial grass	Dry and desert steppes
*Convolvulus ammannii* Desr.	Convolvulaceae	Perennial herb	Debris and stony slopes, sandy and debris desert steppes
*Dontostemon integrifolius* (L.) C. A. Mey.	Brassicaceae	Annual herb	Sandy and debris steppes
*Elymus chinensis* Trin.	Poaceae	Perennial grass	Meadow steppe and alkaline watersides, sands and pebbles, steppe and meadow slopes
*Heteropappus altaicus* (Willd.) Novopokr.	Asteraceae	Perennial herb	Steppes, steppe meadows
*Koeleria cristata* (L.) Pers.	Poaceae	Perennial grass	Meadows, meadow slopes and montane steppes
*Potentilla bifurca* L.	Rosaceae	Perennial herb	Strongly grazed gravelly desert steppe
*Ptilotrichum canescens* (DC.) C. A. Mey.	Brassicaceae	Perennial herb	Stony and debris steppe and desert steppe slopes
*Stipa glareosa* P. Smirn.	Poaceae	Perennial grass	Sandy and debris desert steppes
*Stipa krylovii* Roshev.	Poaceae	Perennial grass	Dry and stony steppes, sandy and debris steppe slopes

### 2.3 Trait measurements

Canopy height was determined in cm as the shortest distance from ground level to the highest photosynthetic tissue (Pérez‐Harguindeguy et al., 2013). For plant width, the diameter (cm) of the maximum distance of the area covered by the foliage when projected on the ground was measured. Five leaves per individual were investigated to determine SLA, defined by Pérez‐Harguindeguy et al. (2013) as the ratio of fresh leaf area to dry mass (mm^2^/mg). For each individual, two leaves were selected to measure chlorophyll fluorescence of the photosystem II (PSII). The measurements were done with a portable continuous excitation time‐resolved chlorophyll fluorimeter (PocketPEA from Hansatec). The *F*_v_/*F*_m_ ratio represents the maximum quantum efficiency of PSII (Maxwell & Johnson, 2000). Beside the *F*_v_/*F*_m_ value, also the performance index, expressed on absorption basis (PI_abs_), is calculated from the chlorophyll fluorescence measurements (Strasser, Srivastava, & Tsimilli‐Michael, 2000). Aboveground biomass of each individual was estimated by clipping at the base of the plant, oven‐drying at 70°C for 48 hr, and weighing at an accuracy of 1 μg. Separation of grass individuals is not straightforward. However, in the case of the tussock grasses *Agropyron cristatum* and *Cleistogenes squarrosa*, we always harvested the whole tussock for biomass measurement. To assess the density and size of stomata, stomatal imprints were made using the clear nail polish method as described by Hilu and Randall (1984). For the imprints, one leaf per individual was collected. Due to the small size of the leaves, one imprint from the abaxial surface and one from the adaxial surface were taken and very hairy leaves were shaved in advance. Four species (*Artemisia frigida*, *Potentilla bifurca*, *Stipa glareosa*, and *Stipa krylovii*) were excluded from the species pool for stomata traits because of hairiness or position of the stomata at the inner layer of small rolled leaves. Stomatal imprints were analyzed using an Olympus CH20 light microscope at a 400‐fold magnification. For each imprint, two fields of view were chosen randomly for counting all visible stomata (stomata density) and measuring guard cell length (stomata size) and width (in μm) for two stomata per field of view. SPI (dimensionless) was calculated as proposed by Sack, Cowan, Jaikumar, and Holbrook (2003) as follows:

(1) $$SPI={\left(guard\;cell\;length\right)}^{2}\times stomatal\;density$$

### 2.4 Statistical analyses

To test whether more species change their trait values in response to grazing with increasing precipitation, we first tested per site and species for differences in trait values between the plots representing different grazing intensity (A–E). For simplicity and as in many cases, data were not normally distributed, nonparametric Kruskal–Wallis tests were used (in total 121 tests = sum of species of all sites). In a second step, per site and trait, we determined the percentage of responding species (i.e., number of species with significant differences based on Kruskal–Wallis tests) using a vote counting approach. In a third step, we analyzed per trait, whether the percentage of responding species changed in response to changing precipitation using linear models with perc_spp_ ~MAP.

Additionally, we tested whether trait values per species differed with changing MAP and grazing intensity. Trait values were defined as the dependent variable, and MAP as well as grazing intensity were used alone and in interaction as explanatory variables (trait ~MAP*grazing).

To test the second hypothesis addressing the grazing‐induced magnitude of trait variation across the precipitation gradient, first, as a measure of ITV, we calculated the CVs per site, plot (=grazing intensity), species, and trait based on eight individuals. The CV was calculated by dividing the standard deviation (across individuals) by the mean. Second, from these five CVs representing grazing‐induced variation in trait values within each site (MAP) we calculated one site‐specific mean CV per trait and species (in total 690 CVs).

We tested whether CVs differed with changing MAP and whether different traits showed different patterns using a linear mixed effect model. CVs were defined as the dependent variable, MAP as explanatory variable and species as random effect (CV ~MAP + (1|Species)). To account for unimodal relationships along MAP, we also added a quadratic term for MAP in the model (CV ~(MAP2 + MAP) + (1|Species)). To support the interpretation of the results, we additionally compared trait‐specific differences in the CVs irrespective of MAP and grazing using nonparametric Kruskal–Wallis tests with subsequent pairwise Wilcoxon tests.

Second, we tested whether different species show different ITV patterns along the MAP gradient and performed the same linear mixed effect model but we used species as random effect instead of traits (CV ~(MAP2 + MAP) + (1|Traits)).

To support interpretation of the ITV (CV) patterns, we explored the distribution of trait and species CVs in multivariate trait space (14 MAP levels × 690 CVs) using a principal component analysis (PCA). To test whether CVs depend on MAP or grazing intensity in this multivariate trait space, MAP and grazing intensity were correlated post hoc with the PCA axes. SPI was excluded for this analysis due to missing data for the species *A. frigida*, *P. bifurca*, *S. glareosa*, and *S. krylovii*.

All analyses were done in R version 3.3.0 (R Core Team, 2016).

## 3 RESULTS

### 3.1 Percentage of responding species along the precipitation gradient

**Figure 2:**
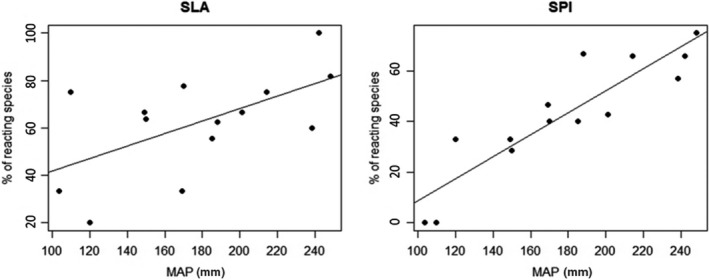
Relation between percentage of investigated species which show significant differences in their trait values across the five different grazing intensities (“% of reacting species”), and the mean annual precipitation (MAP) for the traits specific leaf area (SLA; *R* ^2^ = .35, *F*_1,12_ = 6.46, *p* < .05) and stomatal pore area index (SPI; *R* ^2^ = .40, *F*_1,12_ = 7.94, *p* < .05)

Species‐specific trait responses to grazing under different MAP were highly diverse (Table S1). Only in SLA and SPI, there was an increase in the percentage of species responding to grazing with higher ITV under increasing MAP (SLA: *R* ^2^ = .35, *F*_1,12_ = 6.46, *p* < .05; SPI: *R* ^2^ = .40, *F*_1,12_ = 7.94, *p* < .05; Figure 2). For the other traits (canopy height, plant width, *F*_v_/*F*_m_, PI_abs_, and biomass), models were not significant. It was apparent that grazing and MAP had an interacting effect on SLA in eight of the 15 species. Only *Convolvulus ammanii*, *Elymus chinensis*, and *S. glareosa* did not respond to neither MAP nor grazing with a change in their SLA values. Canopy height showed only for six of 15 species a significant response to the interaction of grazing and MAP. Plant width revealed significant responses to interacting grazing and MAP only for four of 15 species. Plant width of *Chenopodium album*, *Dontostemon integrifolius*, *P. bifurca*, and *Ptilotrichum canescens* did not respond at all. Stomatal traits did only hardly respond grazing intensity; effects of grazing on SPI were only significant in the species *Artemisia adamsii*, *C. ammanii*, *D. integrifolius*, *Koeleria cristata*, and *P. canescens*. MAP and grazing largely affected chlorophyll fluorescence parameters, and 12 of 15 species responded in *F*_v_/*F*_m_ and PI_abs_. The most responsive trait along the gradients was biomass, as all species except for *D. integrifolius* and *P. canescens* changed their biomass in response to MAP and grazing. For linear relationships between trait values and precipitation*grazing, see Table S2.

### 3.2 Variation of traits and species in response to grazing along the precipitation gradient

**Figure 3:**
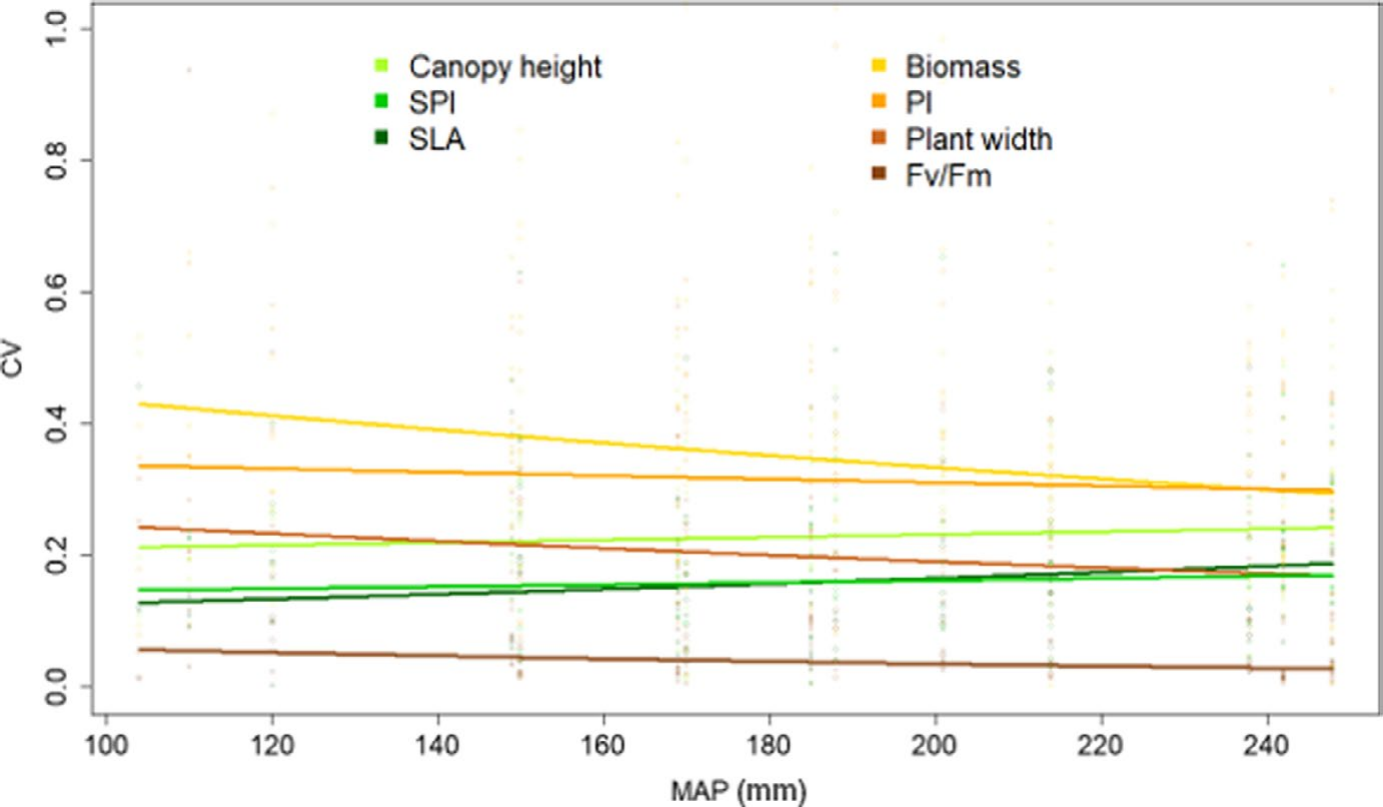
Relation between plot‐wise coefficients of variation of the plant traits canopy height, plant width, specific leaf area (SLA), PI_abs_, *F*_v_/*F*_m_, stomatal pore area index (SPI), and aboveground biomass of the 15 investigated species across the five grazing intensities per mean annual precipitation (MAP) level

The outcome of the model analyzing changes in the magnitude of ITV showed significant relationships with MAP (Table S3), but no consistent patterns of traits (Figure 3). The CVs of canopy height, SPI, and SLA increased with increasing precipitation, whereas the variation of the traits biomass, plant width, PI_abs_, and *F*_v_/*F*_m_ decreased with increasing precipitation.

Irrespective of MAP and grazing, CVs significantly differed between the traits (chi^2^ = 345.02, *df* = 6, *p* < .001; Figure S1). CVs were lowest for *F*_v_/*F*_m_ and highest for biomass and PI_abs_.

**Figure 4:**
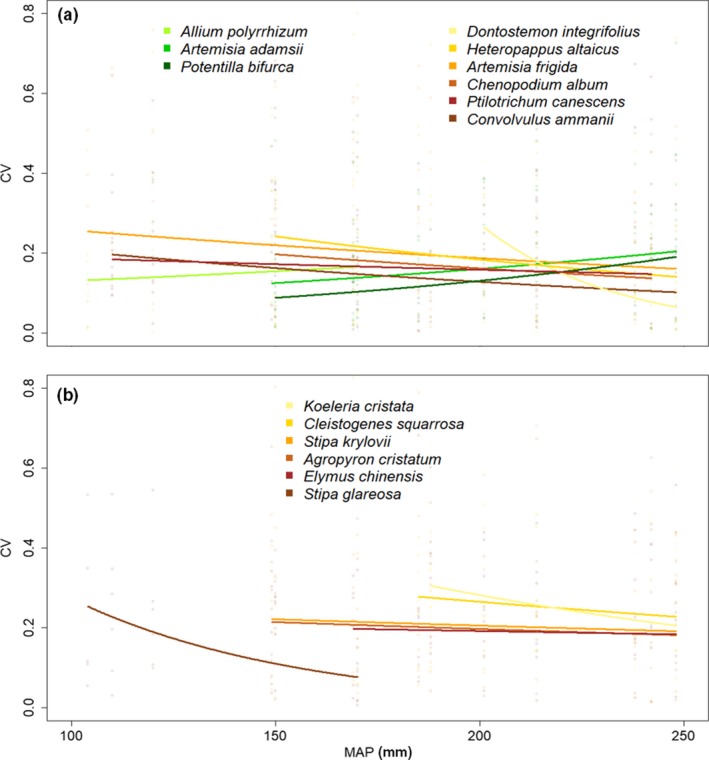
Relation between plot‐wise coefficients of variation of the plant traits canopy height, plant width, specific leaf area (SLA), PI_abs_, *F*_v_/*F*_m_, stomatal pore area index (SPI), and aboveground biomass of the 15 investigated species across the five grazing intensities per mean annual precipitation (MAP) level. (a) Shows coefficients of variation for all investigated herbs, (b) shows coefficients of variation for all investigated grasses

The results of the model analyzing the CVs of the 15 investigated species in response to grazing showed significant, though very weak relationships with MAP (Table S4, Figure 4) and no overall pattern was detected. Twelve out of the 15 species (80%) even revealed higher variation at the drier part of the gradient than at the wetter part, including all examined grass species. Only three species (*Allium polyrrhizum*, *A. adamsii*, and *P. bifurca*) had highest variation caused by grazing under high MAP.

The first axis of the PCA (species per MAP level x grazing‐induced CVs of six traits; Figure S2) explains 26.2% and the second axis 21.0% of the total variation. We found no clear differentiation of the 15 species and their relatedness based on the coefficients of variation of their measured traits. MAP was not significantly correlated with the axes.

## 4 DISCUSSION

This study investigated different aspects of ITV as a response to grazing under increasing MAP. We show that with increasing precipitation, the percentage of species significantly responding to grazing increases for the two traits SLA and SPI, therewith partly confirming our first hypothesis.

Our second hypothesis that the magnitude with which species change their trait values in response to grazing increases with increasing precipitation could be confirmed for three out of seven traits; however, general trends at the species level could not be detected.

### 4.1 Trait value changes in response to grazing with increasing precipitation

Besides the well‐studied effect of grazing, we additionally investigated the influence of MAP on trait values in our study. As hypothesized, we hardly observed grazing‐induced changes in SLA values in the drier part of the precipitation gradient; in contrast, most species showed significant responses to grazing at the wetter part of the gradient. This can be linked to the nonequilibrium concept of rangeland dynamics, which predicts that the potential for grazing‐induced degradation is low in rangelands with relatively low precipitation (Fernandez‐Gimenez & Allen‐Díaz, 1999; von Wehrden, Hanspach, Kaczensky, Fischer, & Wesche, 2012). Accordingly, the concept suggests that precipitation is the main limiting factor for vegetation responses in comparatively dry regions and that grazing hardly influences the vegetation. Assuming that the response of the vegetation is the combined response of the single species, we can expect that plant performance is also hardly influenced by grazing in relative dry regions.

In general, it is assumed that SLA is influenced by changing intensity of grazing and significant differences in SLA values were already found along grazing gradients in other studies (Díaz et al., 2007; Díaz, Noy‐meir, & Cabido, 2001; Zheng, Li, Lan, Ren, & Wang, 2015). However, these changes can be species‐specific and reflect varying plant strategies. SLA has been widely used to predict these plant strategies (Pierce et al., 2017; Westoby, 1999). Previous studies showed that in grazing tolerant species, SLA increases with intensified grazing to compensate for biomass loss by higher growth rates (Rota, Manzano, Carmona, Malo, & Peco, 2017; Strahan et al., 2015). Correspondingly, SLA of grazing tolerant species should be lower on plots with low grazing intensities compared to plots with high grazing intensities (Table S1).

Stomatal pore area index showed a similar pattern, being also in line with our first hypothesis. The nonequilibrium concept of rangeland dynamics can also be applied in this context. The investigated species rarely showed differences in trait values between different grazing intensities at the dry part of the precipitation gradient, but strongly varied between grazing intensities at the wet part of the precipitation gradient. SPI as an integrated measure of stomata size and density can also be used as a proxy for the response to stress (Bucher et al., 2016; Hetherington & Woodward, 2003). In this case, increased SPI indicates increased stress as imposed by continuous grazing, because, for instance, plants try to thicken their leaves as protection under increasing grazing (Westoby, Falster, Moles, Vesk, & Wright, 2002). According to this, more stomata are necessary to provide the supply with CO_2_ also in deeper leaf layers (Körner, Bannister, & Mark, 1986).

Morphological growth traits (canopy height, plant width, individual biomass) are generally highly determined by moisture availability (Lang et al., 2019) but also by prevailing vegetation structure. The largest differences in vegetation structure are mainly found between different steppe types (e.g., desert and meadow steppe) that occur along the precipitation gradient. On this scale, the role of land cover may be overridden by the large‐scale climate effect (Luoto, Virkkala, & Heikkinen, 2007), therefore masking potential grazing effects on this group of traits. However, chlorophyll fluorescence parameters (*F*_v_/*F*_m_, PI_abs_) were independent from grazing intensity in our data set. This observation may be due to the fact that these measures more directly reflect the current status of photosynthetic performance rather than an integrating trait like SPI (integrating for growth conditions over the last months).

### 4.2 Variation of traits and species in response to grazing along the precipitation gradient

Our analysis of the CVs of the seven investigated traits in response to grazing partly confirmed hypothesis (ii), as it was shown that trait variation is significantly dependent on MAP but not in a parallel manner for all investigated traits. The variation of the traits canopy height, SLA, and SPI increased with increasing precipitation, which is in line with our expectations. In general, canopy height was found to be the best predictor to estimate the grazing response of species (Díaz et al., 2001), but still depends on the growth strategy. Consistent with other studies (An & Li, 2014; Díaz et al., 2001), our results show that there is an increasing variation in canopy height between the grazed plots with increasing precipitation. Typically, canopy height decreases with high grazing pressure (Table S2). A common ecological hypothesis for the reduction of the aboveground annual net primary productivity (ANPP) in habitats with low competition for light, such as steppes, is that plants change their growth strategy with long‐term grazing pressure to avoid grazing. Hence, tall plants are better accessible to grazers and are therefore affected more strongly (Falster & Westoby, 2003).

Specific leaf area in grassland species is highly influenced by multiple response strategies and dependent on the site conditions (Díaz et al., 2007; Westoby, 1999). SLA includes leaf mass and leaf size and can be influenced by grazing resistance and avoidance strategies, for example, increase in leaf toughness associated with low palatability (Cornelissen et al., 1999; Grime, Cornelissen, Thompson, & Hodgson, 1996). According to Herms and Mattson, (1992), increased leaf toughness can be negatively correlated with leaf growth, resulting in a reduction of SLA. Additionally, when species react to grazing by reducing leaf size, SLA decreases. Reduced leaf growth also is a strategy of small plants on grazed sites with more bare ground to decrease their capacity for light harvesting, because of too much incoming light. SLA can as well decrease when species try to avoid grazing by developing smaller leaves and are reduced in plant height. In contrast, a strategy of grazing tolerant species is to show bigger leaves with a fast regrowth capacity, tolerating partial defoliation (Briske, 1996; Cingolani et al., 2005; Westoby, 1999).

Stomatal pore area index is estimated by stomatal density and stomatal size. Stomatal densities were found to be sensitive to abiotic environmental conditions (Bucher et al., 2016; Kumekawa et al., 2013; Römermann et al., 2016). High densities of small stomata enable greater and faster stomatal control, which is necessary for plants during drought conditions (Drake, Froend, & Franks, 2013; Franks & Beerling, 2009), for example, at the dry part of our MAP gradient. Additionally, high photosynthesis rates are generated by high maximum leaf diffusive conductance, which is again induced by high densities of small stomata (Drake et al., 2013). Earlier studies detected highest water use efficiency in species with highest stomatal densities (Bucher et al., 2016, 2018; Franks & Beerling, 2009; Franks & Farquhar, 2006). Changes in SPI in our study may be most strongly associated with changes in stomatal density because several studies found stomatal size as closely linked to genome size, with plants possessing larger genomes having larger guard cells (Franks & Beerling, 2009; Jordan, Carpenter, Koutoulis, Price, & Brodribb, 2015).

In spite of these studies, little is known about the relationship between stomata parameters and grazing. Yang, Han, Zhou, and Li (2007) showed in a study about stomata of *Leymus chinensis* that soil water was the first factor for determining stomatal density followed by annual precipitation, which suggests that water availability is the primary ecological factor influencing stomatal density. They found that water use efficiency of *L. chinensis* increased significantly with environmental droughts and was sensitive to the soil water content. The soil water, however, can again be influenced by grazing as high grazing intensity leads to lower vegetation cover and the soil then hardens and dries. Hence, grazing might be influencing stomatal parameters in an indirect manner (Yang et al., 2007).

Contrary to our expectations, the traits plant width, biomass, *F*_v_/*F*_m_, and PI_abs_ showed decreasing variation with increasing precipitation. With the examination of a growth response by biomass, previous studies revealed a decrease of biomass production with grazing, consistent with the changing canopy height in response to grazing (An & Li, 2014). In our study, we found a contrasting effect, but this can again result from the mixture of different species, with variable responses to grazing (Table S2). Less changes in biomass between different grazing intensities under increased precipitation are possibly associated with a change in growth strategies of several species (Peper, Jansen, Pietzsch, & Manthey, 2011; Sasaki et al., 2009). Species being tolerant to grazing with a high regeneration potential or prostrate clonal profiting from bare ground situations by less competition for light at heavily grazed sites might even react positively to grazing. They may build up high biomass values under high grazing intensity. In contrast, grazing sensitive species have of course lower biomass on heavily grazed sites as found for some of our focal species (e.g., *S. krylovii*, Table S2).

Chlorophyll fluorescence measurements (*F*_v_/*F*_m_, PI_abs_) can serve as a tool to estimate the health of the photosynthetic system within the plant leaf by telling the extent to which PSll is damaged by environmental stress (Maxwell & Johnson, 2000; Mohammed, Binder, & Gilles, 1995). Grazing continuously affects plant growth in the highly grazed plots. This stress‐like effect may cause damage of the PSll, measurable by a reduction of *F*_v_/*F*_m_ ratio; *F*_v_/*F*_m_ should thus decrease with increasing grazing intensity (Mohammed et al., 1995; Zhao et al., 2009). Interestingly, we found higher variation of *F*_v_/*F*_m_ and PI_abs_ between different grazing intensities at the drier part of our precipitation gradient (Table S2). Yet, the values were just slightly decreasing up to the wetter end of the gradient. We assume that this pattern is related to the repair and defense mechanisms to overcome damages by photoinhibition (Goh, Ko, Koh, Kim, & Bae, 2012). Lichtenthaler (1998) explained with his stress response concept that stress‐induced repair and adaption mechanisms can lead to a restitution of the previous physiological function or induce the establishment of a new even higher physiological standard than the previous state. According to this concept of stress response, we propose as an explanation of the phenomenon of increased *F*_v_/*F*_m_ variation the following stress response concept: We assume that the high irradiation in the Mongolian steppe led to a slight photoinhibition in general. With the addition of grazing and drought as further stressors, stress avoidance and defense mechanisms are induced to compensate damages by grazing, leading to an increase of the photosynthetic performance.

Irrespective of MAP and grazing, the CVs significantly differed between traits. CVs were lowest for *F*_v_/*F*_m,_ while those of biomass and PI_abs_ were highest. *F*_v_/*F*_m_ seemed to be most stable, because CVs were not only low but also showed the smallest range (Figure S1). In contrast, biomass and PI_abs_ displayed a large range of CVs.

In contrast to our expectations, we did not find a general pattern concerning the CVs along the precipitation gradient. The analysis of the CVs of the 15 investigated species in response to grazing showed that most of the investigated species (80%) had highest variation in the dry region of the precipitation gradient. The *R* ^2^ was, however, very low, and ecological interpretations should therefore be taken with caution.

We did not find an overall pattern of changing variation caused by grazing along the precipitation gradient since the investigated species show different responses. *Agropyron cristatum* and *Heteropappus altaicus* were shown to be sensitive to grazing by reduced aboveground growth (e.g., change in SLA). In contrast, *A. polyrrhizum* and *E. chinensis* were tolerant to grazing with generally induced growth (e.g., change in SLA and biomass), which is also in accordance to the herbivore optimization hypothesis (e.g., Hilbig, 1995; Williamson, Detling, Dodd, & Dyer, 1989). Finally, *C. squarrosa* and *K. cristata* might have switched the growth direction depending on light and open ground access, as indicated by biomass and *F*_v_/*F*_m_. The missing response of *Cleistogenes songorica* and *Convolvulus amannii* can be explained by the fact that these are less accessible to grazers. A common ecological hypothesis in habitats with low competition for light, such as steppes, is that plants change their growth strategy with long‐term grazing pressure to avoid grazing. Hence, usually tall species are better accessible to grazers and are therefore stronger affected (Falster & Westoby, 2003; Painter, Detling, & Steingraeber, 1993). On the other hand, the perennial forb *A. adamsii* is proclaimed to be a grazing resistant weed containing secondary compounds (e.g., essential oils) and being therefore an unpalatable weed for grazers (Fernandez‐Gimenez & Allen‐Díaz, 1999). Sasaki, Okayasu, Jamsran, and Takeuchi (2008) showed additionally that annual unpalatable weeds highly increase in cover in heavily grazed situations. Some plant species are generally avoided by most grazers, but nevertheless consumed when stocking rates are very high and availability of palatable plants is low (Jargalsaikhan, 2013; Tuvshintogtokh & Ariungerel, 2013).

The missing overall pattern of trait responses, but the high responsiveness of the traits in general (Table S2), leads to the assumption that different functional traits may respond to diverse environmental factors. Several studies dealt with this fact and did not find a globally consistent trend in the analysis of these patterns (Albert et al., 2010; Kichenin, Wardle, Peltzer, Morse, & Freschet, 2013), as species can have various or even opposite responses in their traits to an environmental gradient (Helm et al., 2019). Additionally, a given species' performance at large scales (i.e., along the precipitation gradient) depends also on its local distribution pattern and natural selection patterns across the gradient. At smaller scales, that is, within the sites/MAP levels, species response strongly depends on micro‐environmental heterogeneity and/or biotic interactions (Gottfried, Pauli, & Grabherr, 1998). Results of previous studies suggest that some species and traits could be affected by local heterogeneity rather than by environmental gradients between sites within their realized niche (Pescador, Bello, Valladares, & Escudero, 2015).

## 5 CONCLUSION

Our results showed that ITV as a response to grazing increases with increasing precipitation for the two traits SLA and SPI. Other investigated traits did not show clear overall patterns, neither did the examined species. However, similar trait response patterns as for SLA and SPI in terms of grazing and precipitation could be identified for trait–environment relationships in further studies. The results of our study also have implications for the use of community‐weighted mean trait values in vegetation analyses (e.g., Bruelheide et al., 2018), as not only species abundances change in response to changing environmental conditions, but also values of traits considerably change. Including this aspect in further studies will improve our understanding of processes acting within and among communities.

## CONFLICT OF INTEREST

None declared.

## AUTHORS' CONTRIBUTION

Birgit Lang collected data, did the analysis and wrote most of the paper. Julian Ahlborn and Munkhzul Oyunbileg are associated PhD students and were substantially involved in collecting the data during field work, as well as the master student Anna Geiger. Batlai Oyuntsetseg organized field work, collected data in the field, and provided substantial help with determination of species. Christine Römermann, Henrik von Wehrden, and Karsten Wesche designed the study, supervised field, and laboratory work. All co‐authors made leading contributions to the manuscript.

## Data Availability

The data that support the findings of this study are available from the corresponding author, BL, upon reasonable request. All data will be publically available from TRY—Plant Trait Database after publication of this article.
