# Supplementary figures and images for: Grazing effects on intraspecific trait variability vary with changing precipitation patterns in Mongolian rangelands
Source: Ecol Evol. 2019 Dec 26;10(2):678–91. doi: 10.1002/ece3.5895 (PMC6988561; doi:10.1002/ece3.5895)

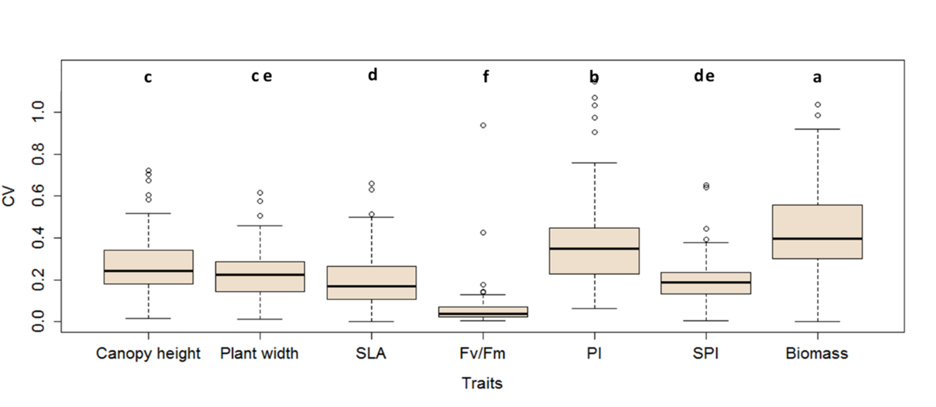

Supplement: Supplementary file 1 [file ECE3-10-678-s001.tif]

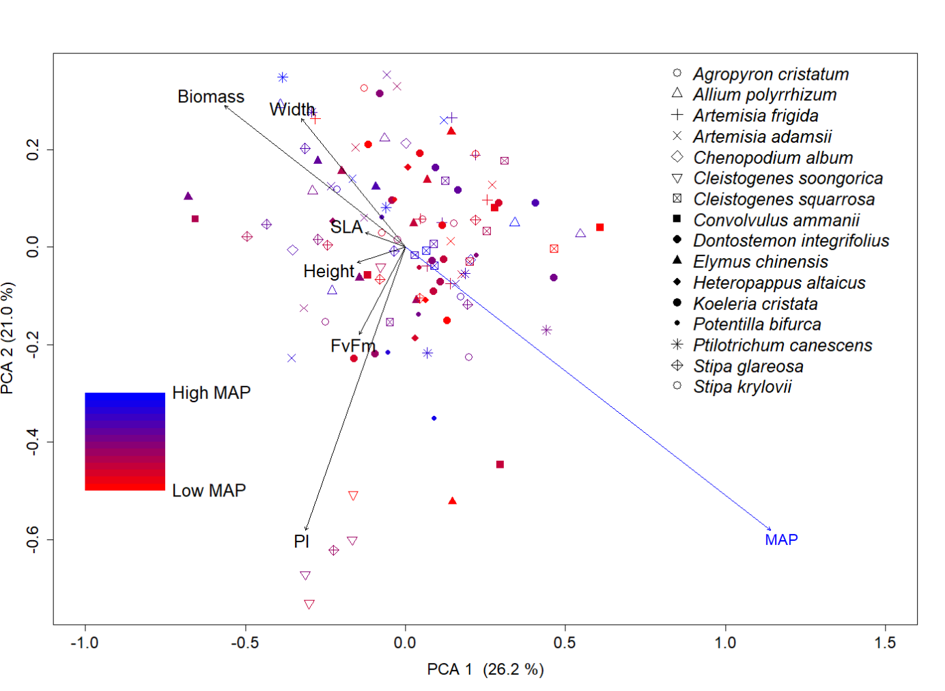

Supplement: Supplementary file 2 [file ECE3-10-678-s002.tif]
