# Supplementary material for: Grazing effects on intraspecific trait variability vary with changing precipitation patterns in Mongolian rangelands
Source: Ecol Evol. 2019 Dec 26;10(2):678–91. doi: 10.1002/ece3.5895 (PMC6988561; doi:10.1002/ece3.5895)
Supplement: Supplementary file 3 [file ECE3-10-678-s003.docx]

**Appendix**

Table S1: Results of the Kruskal-Wallis-tests on the seven traits canopy height, plant width, specific leaf area (SLA), stomatal pore area index (SPI), chlorophyll fluorescence (F_v_/F_m_), performance index (PI_abs_) and aboveground biomass in response to grazing along the precipitation gradient for the 15 investigated species. Given are the means +/- standard error. p < 0.05: *; p < 0.01: **; p < 0.001: ***; n.s.: not significant, NA: data not available.

| Species | MAP [mm] | Canopy height [cm] | | Plant width [cm] | | SLA [mm²/mg] | | SPI | | F_v_/F_m_ | | PI_abs_ | | Biomass [g] | |
| --- | --- | --- | --- | --- | --- | --- | --- | --- | --- | --- | --- | --- | --- | --- | --- |
| *Agropyron cristatum* | 149 | 7.15 ± 0.20 | * | 15.69 ± 0.27 | *** | 8.49 ± 0.23 | *** | 0.13 ± 0.02 | n.s. | 0.74 ± 0.02 | ** | 3.16 ± 0.17 | ** | 0.54 ± 0.10 | *** |
| *Agropyron cristatum* | 150 | 14.33 ± 0.35 | n.s. | 12.90 ± 0.38 | n.s. | 9.16 ± 0.25 | n.s. | 0.17 ± 0.04 | ** | 0.78 ± 0.01 | ** | 4.91 ± 0.27 | ** | 1.36 ± 0.13 | n.s. |
| *Agropyron cristatum* | 170 | 11.60 ± 0.29 | ** | 10.98 ± 0.20 | n.s. | 8.03 ± 0.11 | *** | 0.12 ± 0.02 | n.s. | 0.76 ± 0.01 | *** | 5.78 ± 0.26 | *** | 0.44 ± 0.05 | n.s. |
| *Agropyron cristatum* | 185 | 12.74 ± 0.27 | * | 19.45 ± 0.39 | n.s. | 6.70 ± 0.16 | n.s. | 0.12 ± 0.03 | ** | 0.67 ± 0.03 | *** | 2.22 ± 0.25 | ** | 1.40 ± 0.28 | * |
| *Agropyron cristatum* | 188 | 13.22 ± 0.26 | *** | 17.33 ± 0.26 | *** | 12.38 ± 0.32 | *** | 0.16 ± 0.02 | * | 0.73 ± 0.02 | n.s. | 5.29 ± 0.40 | n.s. | 0.89 ± 0.11 | *** |
| *Agropyron cristatum* | 201 | 7.84 ± 0.14 | ** | 14.10 ± 0.15 | * | 11.54 ± 0.16 | * | 0.13 ± 0.02 | ** | 0.76 ± 0.01 | ** | 5.19 ± 0.19 | * | 0.45 ± 0.09 | * |
| *Agropyron cristatum* | 214 | 10.74 ± 0.39 | *** | 11.22 ± 0.19 | ** | 9.25 ± 0.14 | * | 0.13 ± 0.02 | n.s. | 0.76 ± 0.01 | * | 5.10 ± 0.15 | ** | 0.53 ± 0.09 | ** |
| *Agropyron cristatum* | 238 | 20.61 ± 0.28 | n.s. | 15.09 ± 0.34 | *** | 8.13 ± 0.10 | * | 0.12 ± 0.03 | n.s. | 0.77 ± 0.01 | ** | 4.85 ± 0.18 | *** | 0.50 ± 0.10 | * |
| *Agropyron cristatum* | 242 | 18.36 ± 0.28 | *** | 14.51 ± 0.34 | *** | 12.00 ± 0.20 | * | 0.12 ± 0.02 | n.s. | 0.80 ± 0.00 | *** | 5.15 ± 0.11 | * | 0.67 ± 0.10 | *** |
| *Agropyron cristatum* | 248 | 8.91 ± 0.16 | ** | 9.45 ± 0.17 | n.s. | 7.45 ± 0.17 | *** | 0.15 ± 0.02 | ** | 0.79 ± 0.00 | n.s. | 5.38 ± 0.12 | n.s. | 0.33 ± 0.05 | ** |
| *Allium polyrrhizum* | 104 | 5.01 ± 0.12 | * | 6.46 ± 0.25 | n.s. | 12.46 ± 0.29 | *** | 0.09 ± 0.02 | n.s. | 0.73 ± 0.01 | n.s. | 3.77 ± 0.17 | n.s. | 0.14 ± 0.03 | n.s. |
| *Allium polyrrhizum* | 120 | 6.31 ± 0.31 | *** | 6.67 ± 0.21 | *** | 14.06 ± 0.15 | n.s. | 0.12 ± 0.02 | ** | 0.62 ± 0.02 | * | 1.35 ± 0.14 | n.s. | 0.12 ± 0.06 | *** |
| *Allium polyrrhizum* | 149 | 4.04 ± 0.16 | *** | 7.15 ± 0.19 | * | 10.56 ± 0.28 | *** | 0.12 ± 0.02 | ** | 0.68 ± 0.01 | *** | 2.73 ± 0.20 | *** | 0.08 ± 0.03 | *** |
| *Allium polyrrhizum* | 150 | 8.85 ± 0.20 | * | 5.96 ± 0.30 | n.s. | 14.96 ± 0.31 | * | 0.10 ± 0.02 | n.s. | 0.75 ± 0.01 | n.s. | 3.76 ± 0.28 | n.s. | 0.16 ± 0.07 | n.s. |
| *Allium polyrrhizum* | 169 | 8.03 ± 0.18 | *** | 11.54 ± 0.26 | *** | 11.89 ± 0.18 | n.s. | 0.14 ± 0.02 | * | 0.78 ± 0.01 | * | 6.96 ± 0.18 | * | 0.36 ± 0.07 | *** |
| *Allium polyrrhizum* | 170 | 4.40 ± 0.12 | ** | 5.16 ± 0.16 | ** | 16.62 ± 0.12 | n.s. | 0.08 ± 0.02 | ** | 0.72 ± 0.01 | *** | 3.31 ± 0.17 | *** | 0.07 ± 0.04 | *** |
| *Artemisia adamsii* | 150 | 5.45 ± 0.15 | * | 7.68 ± 0.23 | * | 14.37 ± 0.30 | n.s. | 0.12 ± 0.05 | * | 0.82 ± 0.00 | ** | 14.02 ± 0.21 | n.s. | 0.79 ± 0.14 | *** |
| *Artemisia adamsii* | 169 | 11.41 ± 0.22 | ** | 7.92 ± 0.23 | * | 9.84 ± 0.14 | n.s. | 0.19 ± 0.03 | * | 0.76 ± 0.02 | n.s. | 7.26 ± 0.33 | * | 0.53 ± 0.12 | * |
| *Artemisia adamsii* | 185 | 8.12 ± 0.17 | * | 7.84 ± 0.17 | ** | 8.57 ± 0.08 | n.s. | 0.13 ± 0.02 | *** | 0.76 ± 0.01 | n.s. | 5.85 ± 0.17 | n.s. | 0.32 ± 0.06 | * |
| *Artemisia adamsii* | 201 | 7.34 ± 0.20 | * | 7.82 ± 0.15 | * | 10.47 ± 0.08 | n.s. | 0.18 ± 0.01 | n.s. | 0.77 ± 0.01 | ** | 6.74 ± 0.21 | * | 0.43 ± 0.07 | * |
| *Artemisia adamsii* | 214 | 4.95 ± 0.14 | * | 7.75 ± 0.12 | ** | 10.10 ± 0.31 | *** | 0.16 ± 0.02 | *** | 0.78 ± 0.02 | *** | 12.03 ± 0.26 | *** | 0.36 ± 0.06 | n.s. |
| *Artemisia adamsii* | 238 | 9.25 ± 0.19 | *** | 7.98 ± 0.15 | ** | 10.77 ± 0.08 | ** | 0.14 ± 0.02 | ** | 0.76 ± 0.01 | *** | 5.14 ± 0.16 | ** | 0.41 ± 0.07 | ** |
| *Artemisia adamsii* | 242 | 9.32 ± 0.16 | ** | 6.89 ± 0.18 | *** | 11.65 ± 0.15 | *** | 0.13 ± 0.02 | n.s. | 0.83 ± 0.00 | *** | 12.37 ± 0.13 | *** | 0.52 ± 0.08 | n.s. |
| *Artemisia adamsii* | 248 | 9.21 ± 0.19 | *** | 4.56 ± 0.20 | *** | 11.52 ± 0.28 | *** | 0.15 ± 0.03 | * | 0.80 ± 0.01 | *** | 8.22 ± 0.18 | n.s. | 0.33 ± 0.09 | *** |
| *Artemisia frigida* | 104 | 6.52 ± 0.24 | *** | 11.94 ± 0.22 | *** | 10.61 ± 0.12 | n.s. | NA | NA | 0.81 ± 0.00 | *** | 13.44 ± 0.21 | n.s. | 1.81 ± 0.16 | ** |
| *Artemisia frigida* | 110 | 8.88 ± 0.16 | * | 18.26 ± 0.21 | * | 11.91 ± 0.20 | ** | NA | NA | 0.82 ± 0.01 | ** | 13.70 ± 0.25 | * | 2.73 ± 0.22 | *** |
| *Artemisia frigida* | 120 | 6.17 ± 0.19 | n.s. | 10.76 ± 0.34 | *** | 10.45 ± 0.24 | n.s. | NA | NA | 0.77 ± 0.01 | * | 7.54 ± 0.40 | ** | 1.07 ± 0.17 | n.s. |
| *Artemisia frigida* | 149 | 8.56 ± 0.19 | *** | 16.17 ± 0.20 | * | 10.89 ± 0.28 | *** | NA | NA | 0.76 ± 0.02 | *** | 7.76 ± 0.24 | *** | 0.90 ± 0.09 | ** |
| *Artemisia frigida* | 150 | 13.32 ± 0.24 | *** | 26.55 ± 0.34 | *** | 16.77 ± 0.25 | n.s. | NA | NA | 0.83 ± 0.00 | *** | 16.69 ± 0.28 | *** | 4.88 ± 0.33 | *** |
| *Artemisia frigida* | 169 | 14.06 ± 0.62 | ** | 15.18 ± 0.79 | ** | 15.86 ± 0.41 | ** | NA | NA | 0.81 ± 0.01 | ** | 11.22 ± 0.18 | n.s. | 2.26 ± 0.39 | n.s. |
| *Artemisia frigida* | 170 | 9.94 ± 0.20 | n.s. | 14.10 ± 0.23 | *** | 11.31 ± 0.16 | * | NA | NA | 0.78 ± 0.01 | * | 9.05 ± 0.26 | *** | 1.38 ± 0.14 | *** |
| *Artemisia frigida* | 185 | 14.89 ± 0.33 | *** | 25.63 ± 0.33 | n.s. | 12.50 ± 0.27 | ** | NA | NA | 0.75 ± 0.02 | *** | 8.10 ± 0.38 | *** | 5.14 ± 0.45 | * |
| *Artemisia frigida* | 188 | 23.68 ± 0.58 | *** | 36.13 ± 0.58 | ** | 10.66 ± 0.20 | n.s. | NA | NA | 0.65 ± 0.01 | n.s. | 2.13 ± 0.16 | n.s. | 7.53 ± 0.53 | *** |
| *Artemisia frigida* | 201 | 4.01 ± 0.15 | *** | 10.13 ± 0.19 | * | 15.77 ± 0.38 | *** | NA | NA | 0.77 ± 0.01 | *** | 7.62 ± 0.38 | *** | 0.56 ± 0.10 | * |
| *Artemisia frigida* | 214 | 5.32 ± 0.32 | *** | 10.92 ± 0.21 | ** | 11.22 ± 0.32 | *** | NA | NA | 0.81 ± 0.01 | *** | 17.32 ± 0.26 | ** | 1.03 ± 0.14 | *** |
| *Artemisia frigida* | 238 | 14.99 ± 0.16 | n.s. | 12.85 ± 0.22 | n.s. | 14.59 ± 0.13 | n.s. | NA | NA | 0.81 ± 0.01 | *** | 9.27 ± 0.20 | *** | 0.51 ± 0.08 | n.s. |
| *Artemisia frigida* | 242 | 11.46 ± 0.24 | n.s. | 13.89 ± 0.24 | * | 13.53 ± 0.22 | ** | NA | NA | 0.83 ± 0.00 | *** | 12.60 ± 0.19 | n.s. | 1.09 ± 0.21 | n.s. |
| *Artemisia frigida* | 248 | 10.01 ± 0.31 | *** | 14.24 ± 0.20 | n.s. | 12.59 ± 0.22 | *** | NA | NA | 0.80 ± 0.01 | *** | 7.37 ± 0.16 | *** | 1.06 ± 0.14 | n.s. |
| *Chenopodium album* | 150 | 6.39 ± 0.17 | ** | 4.43 ± 0.19 | * | 12.14 ± 0.22 | *** | 0.18 ± 0.01 | n.s. | 0.79 ± 0.01 | *** | 9.29 ± 0.36 | *** | 0.23 ± 0.08 | *** |
| *Chenopodium album* | 169 | 14.40 ± 0.23 | n.s. | 16.84 ± 0.32 | n.s. | 13.17 ± 0.15 | n.s. | 0.06 ± 0.01 | *** | 0.73 ± 0.01 | * | 3.20 ± 0.13 | n.s. | 2.36 ± 0.35 | n.s. |
| *Chenopodium album* | 214 | 12.70 ± 0.55 | * | 30.16 ± 0.48 | n.s. | 12.35 ± 0.07 | ** | 0.11 ± 0.02 | n.s. | 0.73 ± 0.02 | n.s. | 4.17 ± 0.28 | n.s. | 5.21 ± 0.56 | n.s. |
| *Chenopodium album* | 238 | 6.64 ± 0.18 | *** | 9.18 ± 0.24 | * | 18.39 ± 0.18 | n.s. | 0.17 ± 0.02 | ** | 0.78 ± 0.01 | *** | 4.49 ± 0.17 | ** | 0.33 ± 0.08 | ** |
| *Chenopodium album* | 242 | 7.09 ± 0.18 | * | 7.75 ± 0.23 | *** | 16.04 ± 0.16 | *** | 0.16 ± 0.02 | n.s. | 0.82 ± 0.00 | ** | 7.42 ± 0.18 | *** | 0.35 ± 0.07 | *** |
| *Cleistogenes squarrosa* | 185 | 7.53 ± 0.26 | ** | 10.99 ± 0.26 | * | 8.57 ± 0.18 | n.s. | 0.03 ± 0.02 | n.s. | 0.46 ± 0.07 | ** | 0.56 ± 0.18 | ** | 0.66 ± 0.11 | ** |
| *Cleistogenes squarrosa* | 188 | 7.73 ± 0.22 | *** | 14.22 ± 0.24 | * | 8.48 ± 0.25 | *** | 0.09 ± 0.01 | n.s. | 0.66 ± 0.02 | ** | 3.07 ± 0.38 | *** | 0.87 ± 0.08 | ** |
| *Cleistogenes squarrosa* | 201 | 6.47 ± 0.15 | ** | 12.03 ± 0.20 | * | 22.44 ± 0.12 | n.s. | 0.11 ± 0.01 | *** | 0.63 ± 0.02 | *** | 1.32 ± 0.13 | *** | 0.28 ± 0.07 | ** |
| *Cleistogenes squarrosa* | 214 | 8.16 ± 0.30 | *** | 15.31 ± 0.29 | n.s. | 14.76 ± 0.26 | * | 0.10 ± 0.01 | *** | 0.64 ± 0.02 | *** | 3.28 ± 0.37 | *** | 0.62 ± 0.09 | * |
| *Cleistogenes squarrosa* | 248 | 4.68 ± 0.10 | ** | 8.91 ± 0.12 | * | 18.83 ± 0.26 | *** | 0.11 ± 0.01 | n.s. | 0.71 ± 0.01 | *** | 2.12 ± 0.27 | *** | 0.31 ± 0.07 | * |
| *Convolvulus ammanii* | 110 | 4.97 ± 0.11 | * | 7.07 ± 0.17 | * | 13.65 ± 0.16 | * | 0.18 ± 0.03 | n.s. | 0.77 ± 0.02 | *** | 8.86 ± 0.22 | *** | 0.26 ± 0.05 | ** |
| *Convolvulus ammanii* | 120 | 3.82 ± 0.09 | n.s. | 5.78 ± 0.11 | ** | 14.86 ± 0.14 | n.s. | 0.17 ± 0.02 | * | 0.76 ± 0.01 | n.s. | 6.66 ± 0.26 | ** | 0.17 ± 0.04 | ** |
| *Convolvulus ammanii* | 149 | 3.45 ± 0.12 | ** | 5.61 ± 0.12 | * | 10.11 ± 0.15 | n.s. | 0.18 ± 0.02 | * | 0.78 ± 0.01 | ** | 5.88 ± 0.23 | *** | 0.20 ± 0.04 | *** |
| *Convolvulus ammanii* | 150 | 5.73 ± 0.16 | * | 7.96 ± 0.15 | ** | 15.76 ± 0.23 | ** | 0.14 ± 0.02 | n.s. | 0.78 ± 0.01 | *** | 5.71 ± 0.22 | *** | 0.34 ± 0.05 | *** |
| *Convolvulus ammanii* | 169 | 6.04 ± 0.16 | ** | 9.93 ± 0.14 | * | 9.89 ± 0.14 | *** | 0.15 ± 0.02 | * | 0.81 ± 0.00 | n.s. | 7.66 ± 0.14 | ** | 0.26 ± 0.05 | ** |
| *Convolvulus ammanii* | 170 | 5.33 ± 0.10 | * | 7.50 ± 0.15 | ** | 12.28 ± 0.14 | *** | 0.16 ± 0.02 | * | 0.74 ± 0.02 | *** | 4.34 ± 0.23 | *** | 0.21 ± 0.05 | *** |
| *Convolvulus ammanii* | 188 | 5.41 ± 0.18 | ** | 10.13 ± 0.62 | n.s. | 11.81 ± 0.16 | n.s. | 0.19 ± 0.03 | * | 0.82 ± 0.01 | ** | 13.98 ± 0.34 | n.s. | 0.25 ± 0.05 | ** |
| *Convolvulus ammanii* | 201 | 2.62 ± 0.12 | *** | 7.16 ± 0.10 | n.s. | 15.43 ± 0.23 | ** | 0.15 ± 0.02 | n.s. | 0.75 ± 0.01 | ** | 4.94 ± 0.15 | n.s. | 0.17 ± 0.03 | * |
| *Convolvulus ammanii* | 214 | 3.17 ± 0.18 | *** | 10.46 ± 0.17 | n.s. | 13.02 ± 0.20 | n.s. | 0.15 ± 0.02 | n.s. | 0.79 ± 0.01 | ** | 8.20 ± 0.22 | ** | 0.39 ± 0.06 | * |
| *Convolvulus ammanii* | 248 | 5.72 ± 0.29 | *** | 9.20 ± 0.21 | n.s. | 10.90 ± 0.30 | * | 0.12 ± 0.02 | ** | 0.81 ± 0.01 | n.s. | 6.00 ± 0.25 | n.s. | 0.31 ± 0.09 | n.s. |
| *Dontostemon integrifolius* | 201 | 7.07 ± 0.23 | *** | 8.49 ± 0.27 | *** | 12.11 ± 0.41 | n.s. | 0.07 ± 0.05 | * | 0.66 ± 0.02 | n.s. | 2.03 ± 0.24 | * | 0.20 ± 0.12 | ** |
| *Dontostemon integrifolius* | 214 | 9.86 ± 0.33 | ** | 11.61 ± 0.25 | n.s. | 9.41 ± 0.40 | n.s. | 0.13 ± 0.02 | n.s. | 0.76 ± 0.02 |  | 9.46 ± 0.82 |  | 0.24 ± 0.08 | n.s. |
| *Dontostemon integrifolius* | 238 | 10.19 ± 0.16 | * | 9.09 ± 0.23 | n.s. | 13.27 ± 0.20 | n.s. | 0.18 ± 0.02 | n.s. | 0.77 ± 0.01 | *** | 4.22 ± 0.20 | *** | 0.19 ± 0.07 | ** |
| *Dontostemon integrifolius* | 242 | 7.06 ± 0.13 | * | 7.66 ± 0.17 | *** | 15.25 ± 0.28 | ** | 0.13 ± 0.02 | * | 0.80 ± 0.00 | *** | 4.60 ± 0.17 | n.s. | 0.13 ± 0.04 | n.s. |
| *Dontostemon integrifolius* | 248 | 7.34 ± 0.13 | n.s. | 3.96 ± 0.27 | n.s. | 15.44 ± 0.23 | n.s. | 0.14 ± 0.03 | n.s. | 0.78 ± 0.01 | * | 2.91 ± 0.17 | n.s. | 0.05 ± 0.04 | n.s. |
| *Elymus chinensis* | 169 | 14.92 ± 0.11 | n.s. | 21.32 ± 0.41 | *** | 9.21 ± 0.24 | *** | 0.18 ± 0.03 | ** | 0.80 ± 0.00 | *** | 8.77 ± 0.19 | ** | 0.23 ± 0.04 | ** |
| *Elymus chinensis* | 185 | 13.82 ± 0.23 | ** | 12.96 ± 0.22 | ** | 6.05 ± 0.17 | *** | 0.18 ± 0.03 | n.s. | 0.74 ± 0.01 | *** | 4.79 ± 0.14 | n.s. | 0.17 ± 0.05 | *** |
| *Elymus chinensis* | 188 | 16.12 ± 0.29 | *** | 14.75 ± 0.28 | *** | 7.81 ± 0.20 | *** | 0.24 ± 0.01 | n.s. | 0.73 ± 0.02 | *** | 6.90 ± 0.33 | *** | 0.24 ± 0.04 | *** |
| *Elymus chinensis* | 201 | 12.49 ± 0.17 | *** | 16.61 ± 0.32 | *** | 11.96 ± 0.15 | *** | 0.22 ± 0.02 | n.s. | 0.75 ± 0.01 | *** | 4.41 ± 0.16 | *** | 0.22 ± 0.05 | ** |
| *Elymus chinensis* | 214 | 13.93 ± 0.16 | *** | 14.97 ± 0.19 | * | 12.01 ± 0.29 | *** | 0.28 ± 0.01 | ** | 0.79 ± 0.00 | *** | 6.86 ± 0.16 | *** | 0.29 ± 0.04 | ** |
| *Elymus chinensis* | 238 | 16.46 ± 0.13 | *** | 12.50 ± 0.20 | *** | 6.70 ± 0.23 | *** | 0.21 ± 0.02 | *** | 0.77 ± 0.01 | NA | 5.15 ± 0.11 | NA | 0.24 ± 0.04 | * |
| *Elymus chinensis* | 242 | 15.91 ± 0.15 | *** | 11.66 ± 0.24 | *** | 9.36 ± 0.26 | *** | 0.23 ± 0.02 | n.s. | 0.81 ± 0.00 | ** | 6.70 ± 0.18 | n.s. | 0.15 ± 0.05 | *** |
| *Elymus chinensis* | 248 | 11.53 ± 0.17 | *** | 9.36 ± 0.27 | *** | 8.70 ± 0.19 | *** | 0.25 ± 0.02 | ** | 0.79 ± 0.01 | *** | 4.97 ± 0.15 | ** | 0.16 ± 0.05 | *** |
| *Heteropappus altaicus* | 150 | 12.46 ± 0.39 | * | 24.03 ± 0.43 | n.s. | 12.23 ± 0.30 | ** | 0.28 ± 0.05 | n.s. | 0.82 ± 0.01 | * | 10.82 ± 0.35 | * | 1.28 ± 0.18 | * |
| *Heteropappus altaicus* | 169 | 18.73 ± 0.25 | * | 24.46 ± 0.43 | ** | 9.82 ± 0.13 | n.s. | 0.22 ± 0.05 | n.s. | 0.78 ± 0.01 | n.s. | 7.51 ± 0.30 | * | 2.20 ± 0.31 | ** |
| *Heteropappus altaicus* | 170 | 13.48 ± 0.17 | * | 18.37 ± 0.31 | *** | 12.12 ± 0.23 | *** | 0.26 ± 0.02 | n.s. | 0.77 ± 0.01 | ** | 3.86 ± 0.24 | ** | 1.31 ± 0.27 | *** |
| *Heteropappus altaicus* | 185 | 15.38 ± 0.30 | ** | 24.42 ± 0.33 | *** | 8.24 ± 0.12 | * | 0.18 ± 0.04 | n.s. | 0.73 ± 0.02 | *** | 4.96 ± 0.29 | *** | 2.07 ± 0.16 | *** |
| *Heteropappus altaicus* | 188 | 11.81 ± 0.43 | *** | 22.76 ± 0.35 | n.s. | 10.51 ± 0.17 | ** | 0.18 ± 0.02 | * | 0.77 ± 0.01 | ** | 6.30 ± 0.26 | ** | 2.80 ± 0.21 | * |
| *Heteropappus altaicus* | 201 | 15.99 ± 0.28 | *** | 24.25 ± 0.34 | *** | 13.26 ± 0.23 | n.s. | 0.28 ± 0.02 | n.s. | 0.80 ± 0.01 | ** | 9.14 ± 0.22 | *** | 2.91 ± 0.31 | *** |
| *Heteropappus altaicus* | 214 | 13.27 ± 0.34 | *** | 23.94 ± 0.35 | * | 9.87 ± 0.27 | *** | 0.20 ± 0.02 | n.s. | 0.77 ± 0.01 | *** | 8.09 ± 0.24 | ** | 3.08 ± 0.19 | * |
| *Heteropappus altaicus* | 238 | 15.28 ± 0.17 | * | 13.92 ± 0.23 | * | 10.82 ± 0.21 | ** | 0.29 ± 0.02 | n.s. | 0.82 ± 0.00 | n.s. | 8.50 ± 0.22 | * | 0.85 ± 0.16 | n.s. |
| *Heteropappus altaicus* | 242 | 13.70 ± 0.17 | *** | 16.64 ± 0.25 | ** | 14.31 ± 0.08 | * | 0.20 ± 0.02 | * | 0.84 ± 0.00 | n.s. | 11.48 ± 0.20 | n.s. | 0.83 ± 0.10 | *** |
| *Heteropappus altaicus* | 248 | 6.46 ± 0.58 | n.s. | 10.67 ± 0.25 | n.s. | 12.85 ± 0.21 | n.s. | 0.19 ± 0.05 | n.s. | 0.80 ± 0.03 | * | 7.28 ± 0.46 | * | 0.42 ± 0.10 | n.s. |
| *Koeleria macrantha* | 188 | 10.40 ± 0.23 | * | 20.18 ± 0.25 | n.s. | 6.91 ± 0.35 | ** | 0.08 ± 0.04 | * | 0.68 ± 0.03 | ** | 2.22 ± 0.26 | ** | 1.60 ± 0.16 | *** |
| *Koeleria macrantha* | 238 | 6.48 ± 0.13 | * | 13.10 ± 0.13 | n.s. | 13.59 ± 0.18 | n.s. | 0.13 ± 0.02 | *** | 0.76 ± 0.02 | *** | 5.21 ± 0.22 | * | 0.45 ± 0.06 | ** |
| *Koeleria macrantha* | 242 | 7.86 ± 0.25 | *** | 11.32 ± 0.22 | *** | 12.18 ± 0.16 | *** | 0.10 ± 0.01 | *** | 0.81 ± 0.00 | *** | 6.09 ± 0.15 | * | 0.48 ± 0.08 | ** |
| *Koeleria macrantha* | 248 | 5.40 ± 0.35 | *** | 8.73 ± 0.26 | *** | 11.12 ± 0.27 | *** | 0.14 ± 0.03 | n.s. | 0.76 ± 0.01 | * | 3.24 ± 0.25 | * | 0.17 ± 0.04 | * |
| *Potentilla bifurca* | 150 | 4.94 ± 0.20 | ** | 7.72 ± 0.25 | n.s. | 12.53 ± 0.14 | n.s. | NA | NA | 0.82 ± 0.01 | ** | 12.92 ± 0.28 | n.s. | 0.43 ± 0.11 | n.s. |
| *Potentilla bifurca* | 169 | 3.29 ± 0.09 | * | 5.51 ± 0.09 | * | 8.18 ± 0.12 | n.s. | NA | NA | 0.78 ± 0.01 | * | 6.37 ± 0.15 | * | 0.14 ± 0.04 | *** |
| *Potentilla bifurca* | 170 | 3.35 ± 0.11 | n.s. | 5.56 ± 0.15 | n.s. | 13.02 ± 0.17 | * | NA | NA | 0.78 ± 0.02 | n.s. | 8.24 ± 0.33 | n.s. | 0.17 ± 0.06 | n.s. |
| *Potentilla bifurca* | 185 | 4.00 ± 0.17 | * | 5.59 ± 0.10 | n.s. | 9.55 ± 0.09 | * | NA | NA | 0.79 ± 0.01 | *** | 10.06 ± 0.29 | *** | 0.16 ± 0.06 | ** |
| *Potentilla bifurca* | 201 | 3.09 ± 0.42 | * | 5.25 ± 0.12 | ** | 14.71 ± 0.16 | n.s. | NA | NA | 0.79 ± 0.01 | * | 10.18 ± 0.29 | ** | 0.09 ± 0.03 | *** |
| *Potentilla bifurca* | 214 | 5.99 ± 0.18 | *** | 6.85 ± 0.12 | * | 11.06 ± 0.22 | n.s. | NA | NA | 0.79 ± 0.01 | ** | 10.91 ± 0.27 | *** | 0.23 ± 0.05 | n.s. |
| *Potentilla bifurca* | 238 | 5.27 ± 0.10 | n.s. | 6.10 ± 0.14 | ** | 11.95 ± 0.09 | * | NA | NA | 0.74 ± 0.02 | *** | 5.55 ± 0.30 | *** | 0.20 ± 0.07 | ** |
| *Potentilla bifurca* | 242 | 4.97 ± 0.13 | * | 7.17 ± 0.13 | *** | 11.64 ± 0.13 | * | NA | NA | 0.83 ± 0.00 | *** | 11.05 ± 0.21 | *** | 0.24 ± 0.05 | * |
| *Potentilla bifurca* | 248 | 4.11 ± 0.14 | *** | 5.42 ± 0.12 | *** | 11.99 ± 0.18 | *** | NA | NA | 0.80 ± 0.01 | *** | 6.51 ± 0.21 | *** | 0.18 ± 0.04 | *** |
| *Ptilotrichum canescens* | 110 | 5.17 ± 0.17 | n.s. | 6.21 ± 0.18 | n.s. | 7.77 ± 0.12 | n.s. | NA | NA | 0.72 ± 0.03 | *** | 8.26 ± 0.46 | *** | 0.17 ± 0.03 | n.s. |
| *Ptilotrichum canescens* | 120 | 3.83 ± 0.23 | n.s. | 3.94 ± 0.17 | n.s. | 9.45 ± 0.20 | n.s. | 0.13 ± 0.03 | n.s. | 0.79 ± 0.02 | ** | 14.21 ± 0.47 | * | 0.17 ± 0.07 | ** |
| *Ptilotrichum canescens* | 149 | 7.80 ± 0.00 | * | 4.00 ± 0.00 | ** | 6.08 ± 0.00 | *** | NA | NA | 0.81 ± 0.00 | n.s. | 7.96 ± 0.00 | * | 0.14 ± 0.00 | ** |
| *Ptilotrichum canescens* | 150 | 8.50 ± 0.14 | * | 8.72 ± 0.21 | n.s. | 13.54 ± 0.30 | *** | 0.12 ± 0.04 | n.s. | 0.81 ± 0.01 | *** | 11.89 ± 0.30 | *** | 0.19 ± 0.08 | * |
| *Ptilotrichum canescens* | 170 | 7.33 ± 0.18 | ** | 6.76 ± 0.17 | * | 13.69 ± 0.21 | *** | 0.17 ± 0.04 | n.s. | 0.74 ± 0.02 | ** | 5.91 ± 0.29 | ** | 0.30 ± 0.05 | * |
| *Ptilotrichum canescens* | 185 | 10.47 ± 0.27 | n.s. | 9.93 ± 0.29 | n.s. | 9.27 ± 0.24 | n.s. | 0.13 ± 0.04 | * | 0.78 ± 0.02 | n.s. | 6.95 ± 0.41 | n.s. | 0.16 ± 0.06 | n.s. |
| *Ptilotrichum canescens* | 201 | 8.31 ± 0.31 | *** | 8.31 ± 0.31 | ** | 7.26 ± 0.08 | * | 0.16 ± 0.04 | * | 0.75 ± 0.01 | *** | 6.26 ± 0.36 | *** | 0.23 ± 0.08 | ** |
| *Ptilotrichum canescens* | 214 | 5.42 ± 0.19 | n.s. | 5.46 ± 0.18 | ** | 7.64 ± 0.09 | *** | 0.20 ± 0.04 | n.s. | 0.77 ± 0.01 | ** | 9.67 ± 0.29 | *** | 0.21 ± 0.04 | ** |
| *Ptilotrichum canescens* | 242 | 7.30 ± 0.13 | ** | 7.01 ± 0.20 | ** | 12.83 ± 0.21 | * | 0.19 ± 0.07 | n.s. | 0.83 ± 0.01 | *** | 11.01 ± 0.28 | *** | 0.26 ± 0.07 | ** |
| *Stipa glareosa* | 104 | 8.13 ± 0.15 | n.s. | 11.42 ± 0.49 | ** | 8.22 ± 0.16 | n.s. | NA | NA | 0.77 ± 0.01 | *** | 5.04 ± 0.19 | *** | 0.44 ± 0.08 | *** |
| *Stipa glareosa* | 110 | 11.16 ± 0.16 | *** | 19.30 ± 0.32 | ** | 5.48 ± 0.06 | *** | NA | NA | 0.78 ± 0.01 | *** | 5.45 ± 0.31 | *** | 1.04 ± 0.16 | ** |
| *Stipa glareosa* | 120 | 10.47 ± 0.14 | ** | 16.22 ± 0.22 | *** | 6.24 ± 0.07 | ** | NA | NA | 0.64 ± 0.02 | ** | 2.35 ± 0.29 | *** | 0.34 ± 0.05 | * |
| *Stipa glareosa* | 149 | 10.87 ± 0.17 | ** | 20.67 ± 0.16 | * | 5.47 ± 0.06 | *** | NA | NA | 0.74 ± 0.01 | *** | 3.09 ± 0.17 | *** | 0.84 ± 0.07 | *** |
| *Stipa glareosa* | 150 | 11.07 ± 0.23 | *** | 15.06 ± 0.26 | ** | 7.72 ± 0.13 | *** | NA | NA | 0.75 ± 0.01 | ** | 2.52 ± 0.15 | n.s. | 1.50 ± 0.21 | ** |
| *Stipa glareosa* | 169 | 15.40 ± 0.09 | * | 28.17 ± 0.32 | ** | 5.29 ± 0.11 | *** | NA | NA | 0.80 ± 0.01 | n.s. | 8.75 ± 0.35 | ** | 2.65 ± 0.23 | *** |
| *Stipa glareosa* | 170 | 9.39 ± 0.17 | n.s. | 16.23 ± 0.24 | n.s. | 6.86 ± 0.13 | * | NA | NA | 0.78 ± 0.01 | n.s. | 4.68 ± 0.27 | ** | 0.29 ± 0.06 | n.s. |
| *Stipa krylovii* | 149 | 14.34 ± 0.32 | * | 18.72 ± 0.24 | ** | 6.16 ± 0.07 | n.s. | NA | NA | 0.74 ± 0.01 | *** | 2.76 ± 0.14 | *** | 0.68 ± 0.11 | ** |
| *Stipa krylovii* | 150 | 29.13 ± 0.50 | *** | 22.43 ± 0.33 | *** | 9.27 ± 0.27 | *** | NA | NA | 0.77 ± 0.01 | *** | 3.63 ± 0.20 | *** | 1.76 ± 0.23 | *** |
| *Stipa krylovii* | 169 | 45.17 ± 0.51 | *** | 40.75 ± 0.66 | ** | 7.58 ± 0.24 | n.s. | NA | NA | 0.70 ± 0.02 | ** | 2.25 ± 0.22 | *** | 5.44 ± 0.50 | *** |
| *Stipa krylovii* | 170 | 24.61 ± 0.38 | *** | 15.72 ± 0.23 | ** | 8.03 ± 0.06 | n.s. | NA | NA | 0.75 ± 0.01 | *** | 2.46 ± 0.14 | *** | 0.48 ± 0.05 | n.s. |
| *Stipa krylovii* | 185 | 23.63 ± 0.29 | ** | 31.51 ± 0.21 | n.s. | 7.27 ± 0.11 | ** | NA | NA | 0.70 ± 0.02 | *** | 2.60 ± 0.18 | *** | 2.88 ± 0.28 | *** |
| *Stipa krylovii* | 188 | 25.65 ± 0.35 | *** | 43.39 ± 0.30 | *** | 7.73 ± 0.14 | n.s. | NA | NA | 0.76 ± 0.02 | *** | 6.87 ± 0.45 | *** | 6.54 ± 0.26 | *** |
| *Stipa krylovii* | 201 | 15.90 ± 0.36 | *** | 28.23 ± 0.37 | ** | 8.18 ± 0.11 | ** | NA | NA | 0.74 ± 0.01 | *** | 4.45 ± 0.25 | *** | 3.98 ± 0.67 | ** |
| *Stipa krylovii* | 214 | 20.29 ± 0.24 | ** | 40.86 ± 0.34 | ** | 8.13 ± 0.11 | * | NA | NA | 0.72 ± 0.01 | *** | 3.39 ± 0.20 | *** | 7.25 ± 0.37 | *** |
| *Stipa krylovii* | 238 | 38.72 ± 0.28 | * | 31.19 ± 0.26 | * | 7.59 ± 0.13 | ** | NA | NA | 0.76 ± 0.01 | *** | 3.85 ± 0.16 | *** | 1.94 ± 0.09 | ** |
| *Stipa krylovii* | 242 | 39.10 ± 0.38 | *** | 22.84 ± 0.21 | n.s. | 10.89 ± 0.18 | * | NA | NA | 0.78 ± 0.00 | ** | 4.17 ± 0.20 | *** | 1.29 ± 0.19 | * |
| *Stipa krylovii* | 248 | 18.79 ± 0.28 | *** | 21.84 ± 0.27 | * | 6.95 ± 0.22 | *** | NA | NA | 0.72 ± 0.01 | ** | 1.78 ± 0.16 | * | 1.21 ± 0.10 | * |

Table S2: Results of linear models for changes in the seven traits canopy height, plant width, specific leaf area (SLA), stomatal pore area index (SPI), chlorophyll fluorescence (F_v_/F_m_), performance index (PI_abs_) and aboveground biomass in response to the interaction of mean annual precipitation (MAP) and grazing (Gra) for the fifteen target species. Given are results (R², test statistics and estimates for intercept, MAP, grazing and MAP*grazing) of the minimal adequate model (p < 0.1: .; p < 0.05: *; p < 0.01: **; p < 0.001: ***; n.s.: not significant, NA: data not available).

|  |  | **Canopy height [cm]** | | | **Plant width [cm]** | | | **SLA [mm²/mg]** | | | **SPI** | | | **F_v_/F_m_** | | | **PI_abs_** | | | **Biomass [g]** | | |
| --- | --- | --- | --- | --- | --- | --- | --- | --- | --- | --- | --- | --- | --- | --- | --- | --- | --- | --- | --- | --- | --- | --- |
| *Agropyron  cristatum* |  | R² = | 0.23 |  | R² = | 0.07 |  | R² = | 0.05 |  | R² = | 0.01 |  | R² = | 0.06 |  | R² = | 0.11 |  | R² = | 0.15 |  |
|  |  | F_2,379_ = | 56.02 | *** | F_3,378_ = | 10.01 | *** | F_3,378_ = | 6.32 | *** | F_3_,_306_ = | 0.78 | n.s. | F_2,379_ = | 11.88 | *** | F_3,378_ = | 16.21 | *** | F_2,375_ = | 32.88 | *** |
|  | Estimate | Intercept | 1.166000 | *** | Intercept | 3.574000 |  | Intercept | 2.517000 | *** |  |  |  | Intercept | 0.681800 | *** | Intercept | 1.498000 | *** | Intercept | 0.200000 |  |
|  |  | MAP | 0.004588 | *** | MAP | -0.005561 |  | MAP | -0.001299 |  |  |  |  | MAP | 0.000427 | ** | MAP | 0.000423 |  | MAP | -0.005738 | *** |
|  |  | Gra | 0.000455 | *** | Gra | -0.000777 |  | Gra | -0.000655 | ** |  |  |  | Gra | -0.000012 | . | Gra | -0.001278 | *** | Gra | 0.000395 | *** |
|  |  |  |  |  | MAP*Gra | 0.000005 |  | MAP*Gra | 0.000003 | * |  |  |  |  |  |  | MAP*Gra | 0.000005 | ** |  |  |  |
| *Allium  polyrrhizum* |  | R² = | 0.10 |  | R² = | 0.10 |  | R² = | 0.13 |  | R² = | 0.04 |  | R² = | 0.10 |  | R² = | 0.09 |  | R² = | 0.11 |  |
|  |  | F_2,245_ = | 13.03 | *** | F_1,246_ = | 28.84 | *** | F_3,216_ = | 11.00 | *** | F_3_,_200_ = | 2.81 | n.s. | F_1,230_ = | 25.67 | *** | F_1,230_ = | 22.57 | *** | F_2,243_ = | 14.63 | *** |
|  | Estimate | Intercept | 0.659000 | ** | Intercept | -2.155990 |  | Intercept | -0.187200 |  |  |  |  | Intercept | 0.593217 | *** | Intercept | -0.201372 |  | Intercept | -4.261666 | *** |
|  |  | MAP | 0.006096 | *** | MAP | 0.073950 | *** | MAP | 0.084930 | *** |  |  |  | MAP | 0.000876 | *** | MAP | 0.008925 | *** | MAP | 0.013300 | *** |
|  |  | Gra | 0.000229 | ** |  |  |  | Gra | 0.019360 | *** |  |  |  |  |  |  |  |  |  | Gra | 0.000423 | ** |
|  |  |  |  |  |  |  |  | MAP*Gra | -0.000124 | *** |  |  |  |  |  |  |  |  |  |  |  |  |
| *Artemisia  adamsii* |  | R² = | 0.13 |  | R² = | 0.05 |  | R² = | 0.10 |  | R² = | 0.09 |  | R² = | 0.020000 |  | R² = | 0.04 |  | R² = | 0.10 |  |
|  |  | F_2,302_ = | 13.03 | *** | F_2,302_ = | 8.88 | *** | F_3,298_ = | 11.54 | *** | F_3,242_ = | 7.66 | *** | F_3,301_ = | 1.56 |  | F_3,301_ = | 4.55 | *** | F_3,296_ = | 11.50 | *** |
|  | Estimate | Intercept | 1.642000 | *** | Intercept | 10.547518 | *** | Intercept | 13.840000 | *** | Intercept | 0.027550 |  | Intercept | -0.179300 | *** | Intercept | 3.022000 | *** | Intercept | 0.235700 |  |
|  |  | MAP | 0.000949 |  | MAP | -0.017926 | *** | MAP | -0.011070 |  | MAP | 0.000560 | *** | MAP | -0.000312 |  | MAP | -0.004635 | ** | MAP | -0.007142 | *** |
|  |  | Gra | 0.000304 | *** | Gra | 0.000913 | ** | Gra | -0.012000 | *** | Gra | 0.000121 | ** | Gra | -0.000144 | * | Gra | -0.001488 | *** | Gra | -0.000692 |  |
|  |  |  |  |  |  |  |  | MAP*Gra | 0.000048 | *** | MAP*Gra | -0.000001 | ** | MAP*Gra | 0.000001 | * | MAP*Gra | 0.000007 | *** | MAP*Gra | 0.000005 | * |
| *Artemisia  frigida* |  | R² = | 0.08 |  | R² = | 0.06 |  | R² = | 0.03 |  | NA |  |  | R² = | 0.07 |  | R² = | 0.03 |  | R² = | 0.11 |  |
|  |  | F_3,459_ = | 12.98 | *** | F_2,460_ = | 14.99 | *** | F_2,459_= | 7.26 | *** |  |  |  | F_3,459_ = | 11.64 | *** | F_1,461_ = | 16.15 | *** | F_2,446_ = | 27.34 | *** |
|  | Estimate | Intercept | 2.060000 | *** | Intercept | 1.681000 | *** | Intercept | 10.471893 | *** |  |  |  | Intercept | 0.064800 |  | Intercept | 2.337000 | *** | Intercept | 0.899100 | *** |
|  |  | MAP | -0.000288 |  | MAP | 0.001843 | ** | MAP | 0.013786 | ** |  |  |  | MAP | -0.001274 | *** |  |  |  | MAP | -0.005395 | *** |
|  |  | Gra | -0.000347 |  | Gra | 0.000285 | *** | Gra | -0.001010 | * |  |  |  | Gra | -0.000303 | *** | Gra | -0.000256 | *** | Gra | 0.000413 | *** |
|  |  | MAP*Gra | 0.000004 | ** |  |  |  |  |  |  |  |  |  | MAP*Gra | 0.000001 | ** |  |  |  |  |  |  |
| *Chenopodium  album* |  | R² = | 0.04 |  | R² = | 0.01 |  | R² = | 0.12 |  | R² = | 0.02 |  | R² = | 0.09 |  | R² = | 0.04 |  | R² = | 0.05 |  |
|  |  | F_1,208_ = | 9.23 | ** | F_3,206_ = | 0.98 | n.s. | F_3,206_ = | 8.95 | *** | F_1,208_ = | 4.55 | *** | F_1,208_ = | 20.86 | *** | F_2,207_ = | 4.56 | *** | F_2,207_ = | 5.69 | *** |
|  | Estimate | Intercept | 2.632966 | *** |  |  |  | Intercept | 17.550000 | *** | Intercept | 0.813479 | *** | Intercept | -0.395219 | *** | Intercept | 0.913662 | ** | Intercept | 0.482033 |  |
|  |  | MAP | -0.002984 | ** |  |  |  | MAP | -0.015020 |  | MAP | 0.003316 | * | MAP | 0.000675 | *** | MAP | 0.003428 | * | MAP | -0.004743 | . |
|  |  |  |  |  |  |  |  | Gra | -0.017010 | *** |  |  |  |  |  |  | Gra | -0.000255 | * | Gra | -0.000503 | ** |
|  |  |  |  |  |  |  |  | MAP*Gra | 0.000088 | *** |  |  |  |  |  |  |  |  |  |  |  |  |

| *Cleistogenes  squarrosa* |  | R² = | 0.36 |  | R² = | 0.04 |  | R² = | 0.27 |  | R² = | 0.16 |  | R² = | 0.24 |  | R² = | 0.21 |  | R² = | 0.14 |  |
| --- | --- | --- | --- | --- | --- | --- | --- | --- | --- | --- | --- | --- | --- | --- | --- | --- | --- | --- | --- | --- | --- | --- |
|  |  | F_2,168_ = | 47.37 | *** | F_1,169_ = | 12.07 | ** | F_3,167_ = | 21.10 | *** | F_1,153_ = | 29.03 | *** | F_3,166_ = | 17.54 | *** | F_3,166_ = | 14.45 | *** | F_2,168_ = | 13.92 | *** |
|  | Estimate | Intercept | 2.610366 | *** | Intercept | 3.045035 | *** | Intercept | 14.350000 | * | Intercept | 0.009089 |  | Intercept | 0.214400 |  | Intercept | 5.076000 | *** | Intercept | 1.124448 | . |
|  |  | MAP | 0.005070 | *** | MAP | -0.005811 | ** | MAP | 0.155800 | *** | MAP | 0.000518 | *** | MAP | 0.000988 |  | MAP | 0.023730 | *** | MAP | -0.010893 | *** |
|  |  | Gra | 0.000418 | *** |  |  |  | Gra | 0.010690 |  |  |  |  | Gra | 0.001537 | *** | Gra | 0.009514 | *** | Gra | 0.000317 | ** |
|  |  |  |  |  |  |  |  | MAP*Gra | -0.000072 | * |  |  |  | MAP*Gra | 0.000007 | *** | MAP*Gra | 0.000045 | *** |  |  |  |
| *Convolvulus  ammanii* |  | R² = | 0.05 |  | R² = | 0.10 |  | R² = | 0.01 |  | R² = | 0.10 |  | R² = | 0.08 |  | R² = | 0.04 |  | R² = | 0.01 |  |
|  |  | F_3,364_ = | 6.28 | *** | F_2,365_ = | 19.94 | *** | F_3,362_ = | 1.24 | n.s. | F_2,214_ = | 11.31 | *** | F_3,363_ = | 11.00 | *** | F_1,365_ = | 13.82 | *** | F_1,364_ = | 5.31 | * |
|  | Estimate | Intercept | 1.283000 | *** | Intercept | 3.741265 | *** |  |  |  | Intercept | 0.207200 | *** | Intercept | 0.204200 | *** | Intercept | 1.897000 | ** | Intercept | -1.927551 | *** |
|  |  | MAP | 0.000722 |  | MAP | 0.026907 | *** |  |  |  | MAP | 0.000272 | *** | MAP | 0.000176 |  |  |  |  | MAP | 0.002042 | * |
|  |  | Gra | 0.000868 | ** | Gra | -0.000713 | * |  |  |  | Gra | 0.000010 | * | Gra | 0.000169 | *** | Gra | 0.000284 | ** |  |  |  |
|  |  | MAP*Gra | 0.000005 | ** |  |  |  |  |  |  |  |  |  | MAP*Gra | 0.000001 | ** |  |  |  |  |  |  |
| *Dontostemon  integrifolius* |  | R² = | 0.09 |  | R² = | 0.03 |  | R² = | 0.41 |  | R² = | 0.29 |  | R² = | 0.48 |  | R² = | 0.27 |  | R² = | 0.01 |  |
|  |  | F_3,134_ = | 4.37 | ** | F_3,134_ = | 1.41 | n.s. | F_1,118_ = | 83.43 | *** | F_2,214_ = | 11.96 | *** | F_3,116_ = | 36.17 | *** | F_3,116_ = | 14.46 | *** | F_3,133_ = | 0.38 | n.s. |
|  | Estimate | Intercept | 2.176000 | ** |  |  |  | Intercept | -1.139579 |  | Intercept | 0.532200 | ** | Intercept | 1.558000 | *** | Intercept | 7.680000 | *** |  |  |  |
|  |  | MAP | 0.000709 |  |  |  |  | MAP | 0.003715 | ** | MAP | 0.002893 | ** | MAP | 0.005553 | *** | MAP | 0.038630 | *** |  |  |  |
|  |  | Gra | 0.002129 | * |  |  |  |  |  |  | Gra | 0.000633 | * | Gra | 0.000751 | *** | Gra | 0.009021 | *** |  |  |  |
|  |  | MAP*Gra | 0.000009 | * |  |  |  |  |  |  | MAP*Gra | 0.000003 | * | MAP*Gra | 0.000003 | *** | MAP*Gra | 0.000040 | *** |  |  |  |
| *Elymus  chinensis* |  | R² = | 0.19 |  | R² = | 0.19 |  | R² = | 0.01 |  | R² = | 0.04 |  | R² = | 0.12 |  | R² = | 0.11 |  | R² = | 0.05 |  |
|  |  | F_1,326_ = | 77.03 | ** | F_3,324_ = | 25.76 | *** | F_3,315_ = | 1.36 | n.s. | F_1,307_ = | 12.47 | *** | F_3,323_ = | 15.24 | *** | F_1,325_ = | 41.33 | *** | F_2,324_ = | 7.98 | *** |
|  | Estimate | Intercept | 2.460000 | *** | Intercept | 23.190000 | *** |  |  |  | Intercept | 0.138298 | *** | Intercept | 0.179500 | ** | Intercept | 1.908000 | *** | Intercept | -0.893900 | *** |
|  |  |  |  |  | MAP | -0.046860 | * |  |  |  | MAP | 0.000432 | *** | MAP | 0.000216 |  |  |  |  | MAP | -0.004143 | *** |
|  |  | Gra | 0.000268 | *** | Gra | 0.018850 | *** |  |  |  |  |  |  | Gra | 0.000284 | *** | Gra | 0.000427 | *** | Gra | 0.000102 | . |
|  |  |  |  |  | MAP*Gra | -0.000082 | *** |  |  |  |  |  |  | MAP*Gra | 0.000001 | ** |  |  |  |  |  |  |
| *Heteropappus  altaicus* |  | R² = | 0.16 |  | R² = | 0.04 |  | R² = | 0.06 |  | R² = | 0.01 |  | R² = | 0.05 |  | R² = | 0.04 |  | R² = | 0.05 |  |
|  |  | F_1,343_ = | 66.58 | *** | F_1,343_ = | 12.87 | *** | F_2,343_ = | 11.88 | ** | F_3,223_ = | 1.07 | n.s. | F_2,342_ = | 11.88 | *** | F_1,343_ = | 16.22 | *** | F_2,340_ = | 9.40 | *** |
|  | Estimate | Intercept | 2.251000 | *** | Intercept | 54.694150 | *** | Intercept | -0.344300 | *** |  |  |  | Intercept | 0.344300 | ** | Intercept | 0.653826 | * | Intercept | 0.842761 | * |
|  |  |  |  |  | MAP | -0.163090 | *** | MAP | 0.000591 | * |  |  |  | MAP | 0.000591 | *** | MAP | 0.005844 | *** | MAP | -0.004868 | * |
|  |  | Gra | 0.000452 | *** |  |  |  | Gra | -0.000026 | ** |  |  |  | Gra | 0.000026 | ** |  |  |  | Gra | 0.000362 | ** |
| *Koeleria  cristata* |  | R² = | 0.49 |  | R² = | 0.44 |  | R² = | 0.26 |  | R² = | 0.16 |  | R² = | 0.22 |  | R² = | 0.18 |  | R² = | 0.45 |  |
|  |  | F_2,126_ = | 59.76 | *** | F_3,125_ = | 33.06 | *** | F_2,126_ = | 21.95 | *** | F_3,104_ = | 6.74 | *** | F_3,125_ = | 11.83 | *** | F_1,127_ = | 28.27 | *** | F_3,125_ = | 34.33 | *** |
|  | Estimate | Intercept | 8.482127 | *** | Intercept | -0.462900 |  | Intercept | -0.476969 |  | Intercept | 0.199800 | *** | Intercept | 0.457000 | *** | Intercept | 2.153027 | ** | Intercept | -2.325000 | ** |
|  |  | MAP | 0.017623 | . | MAP | 0.047040 | * | MAP | 0.059720 | *** | MAP | 0.000290 |  | MAP | 0.000833 |  | MAP | 0.014697 | *** | MAP | 0.004171 |  |
|  |  | Gra | 0.004518 | *** | Gra | 0.045910 | *** | Gra | -0.002820 | *** | Gra | 0.000231 | *** | Gra | 0.000459 | ** |  |  |  | Gra | 0.006854 | *** |
|  |  |  |  |  | MAP*Gra | -0.000187 | *** |  |  |  | MAP*Gra | 0.000001 | ** | MAP*Gra | 0.000002 | ** |  |  |  | MAP*Gra | -0.000027 | *** |
| *Potentilla  bifurca* |  | R² = | 0.11 |  | R² = | 0.01 |  | R² = | 0.09 |  | NA |  |  | R² = | 0.08 |  | R² = | 0.10 |  | R² = | 0.03 |  |
|  |  | F_3,316_ = | 13.03 | *** | F_3,316_ = | 1.40 | n.s. | F_3,316_ = | 10.17 | *** |  |  |  | F_3,316_ = | 11.20 | *** | F_2,317_ = | 16.80 | *** | F_3,316_ = | 3.41 | * |
|  | Estimate | Intercept | 1.260000 | *** |  |  |  | Intercept | 10.480000 | *** |  |  |  | Intercept | 0.132600 | ** | Intercept | 3.401000 | *** | Intercept | -2.171000 | *** |
|  |  | MAP | 0.000027 | * |  |  |  | MAP | 0.004889 |  |  |  |  | MAP | 0.000508 | * | MAP | 0.006006 | *** | MAP | 0.001381 |  |
|  |  | Gra | 0.000734 |  |  |  |  | Gra | -0.008313 | *** |  |  |  | Gra | 0.000139 | * | Gra | 0.000214 | ** | Gra | 0.001355 | * |
|  |  | MAP*Gra | 0.000005 | ** |  |  |  | MAP*Gra | 0.000037 | ** |  |  |  | MAP*Gra | 0.000001 | * |  |  |  | MAP*Gra | -0.000006 | * |

| *Ptilotrichum  canescens* |  | R² = | 0.11 |  | R² = | 0.06 |  | R² = | 0.08 |  | R² = | 0.30 |  | R² = | 0.06 |  | R² = | 0.05 |  | R² = | 0.02 |  |
| --- | --- | --- | --- | --- | --- | --- | --- | --- | --- | --- | --- | --- | --- | --- | --- | --- | --- | --- | --- | --- | --- | --- |
|  |  | F_3,256_ = | 13.03 | *** | F_1,257_ = | 1.38 | ns | F_3,256_ = | 0.08 | *** | F3,94 = | 13.55 | *** | F_3,254_ = | 5.61 | *** | F_3,254_ = | 1.20 | ns | F_1,257_ = | 5.97 | * |
|  | Estimate | Intercept | 8.354000 | *** |  |  |  | Intercept | 17.270000 | *** | Intercept | 0.117700 | * | Intercept | 0.198700 | ** |  |  |  | Intercept | -2.098027 | *** |
|  |  | MAP | 0.006846 |  |  |  |  | MAP | -0.031040 | * | MAP | 0.001721 | *** | MAP | 0.000222 |  |  |  |  | MAP | 0.009654 | * |
|  |  | Gra | 0.004055 | * |  |  |  | Gra | -0.009286 | *** | Gra | 0.000231 | *** | Gra | 0.000186 | ** |  |  |  |  |  |  |
|  |  | MAP*Gra | 0.000023 | * |  |  |  | MAP*Gra | 0.000043 | ** | MAP*Gra | 0.000001 | *** | MAP*Gra | 0.000001 | * |  |  |  |  |  |  |
| *Stipa  glareosa* |  | R² = | 0.24 |  | R² = | 0.06 |  | R² = | 0.02 |  | NA |  |  | R² = | 0.01 |  | R² = | 0.01 |  | R² = | 0.18 |  |
|  |  | F_2,211_ = | 33.72 | *** | F_1,212_ = | 14.82 | *** | F_3,210_ = | 1.40 | ns |  |  |  | F_3,209_ = | 0.81 | ns | F_3,209_ = | 0.45 | ns | F_2,209_ = | 22.67 | *** |
|  | Estimate | Intercept | 1.697000 | *** | Intercept | 1.979874 | *** |  |  |  |  |  |  |  |  |  |  |  |  | Intercept | -2.178835 | *** |
|  |  | MAP | 0.003036 | *** | MAP | 0.005622 | *** |  |  |  |  |  |  |  |  |  |  |  |  | MAP | 0.000516 | *** |
|  |  | Gra | 0.000311 | ** |  |  |  |  |  |  |  |  |  |  |  |  |  |  |  | Gra | 0.000546 | *** |
| *Stipa  krylovii* |  | R² = | 0.16 |  | R² = | 0.06 |  | R² = | 0.02 |  | NA |  |  | R² = | 0.01 |  | R² = | 0.02 |  | R² = | 0.06 |  |
|  |  | F_3,428_ = | 27.44 | *** | F_3,428_ = | 9.45 | *** | F_3,427_ = | 3.48 | *** |  |  |  | F_3,428_ = | 1.46 | ns | F_3,428_ = | 2.35 | ns | F_2,421_ = | 14.48 | *** |
|  | Estimate | Intercept | 1.575000 | *** | Intercept | 9.352000 |  | Intercept | 3.869000 | ** |  |  |  |  |  |  |  |  |  | Intercept | -0.966085 | ** |
|  |  | MAP | 0.006440 | *** | MAP | 0.079840 | ** | MAP | 0.020130 | ** |  |  |  |  |  |  |  |  |  | MAP | 0.005325 | ** |
|  |  | Gra | 0.001227 | *** | Gra | 0.019860 | ** | Gra | 0.003377 | * |  |  |  |  |  |  |  |  |  | Gra | 0.000470 | *** |
|  |  | MAP*Gra | 0.000004 | * | MAP*Gra | -0.000072 | * | MAP*Gra | -0.000016 | * |  |  |  |  |  |  |  |  |  |  |  |  |

Table S3: Results of linear mixed effect models for changes in CVs of the seven traits canopy height, plant width, specific leaf area (SLA), stomatal pore area index (SPI), chlorophyll fluorescence (F_v_/F_m_), performance index (PI_abs_) and aboveground biomass in response to mean annual precipitation (MAP). Given are results (Rm², Rc², test statistics and estimates for intercept and MAP) of the minimal adequate model (p < 0.1: .; p < 0.05: *; p < 0.01: **; p < 0.001: ***). Rm² = marginal R²; Rc² = conditional R².

|  | **Canopy height [cm]** | | | **Plant width [cm]** | | | **SLA [mm²/mg]** | | | **SPI** | | | **F_v_/F_m_** | | | **PI_abs_** | | | **Biomass [g]** | | |
| --- | --- | --- | --- | --- | --- | --- | --- | --- | --- | --- | --- | --- | --- | --- | --- | --- | --- | --- | --- | --- | --- |
|  | Rm² = | 0 |  | Rm² = | 0.29 |  | Rm² = | 0 |  | Rm² = | 0.01 |  | Rm² = | 0.05 |  | Rm² = | 0.01 |  | Rm² = | 0.02 |  |
|  | Rc² = | 0.09 |  | Rc² = | 0.07 |  | Rc² = | 0.06 |  | Rc² = | 0.03 |  | Rc² = | 0.05 |  | Rc² = | 0.41 |  | Rc² = | 0.05 |  |
|  | Chi² = | 0.12 |  | Chi² = | 0.41 |  | Chi² = | 0.32 |  | Chi² = | 0.75 |  | Chi² = | 1.00 |  | Chi² = | 0.000 |  | Chi² = | 0.51 |  |
| Estimate | Intercept | 0.268 | *** | Intercept | 0.244 | *** | Intercept | 0.206 | *** | Intercept | 0.193 | *** | Intercept | 0.057 | *** | Intercept | 0.380 | *** | Intercept | 0.429 | *** |
|  | MAP | -0.033 |  | MAP | -0.027 | . | MAP | 0.006 |  | MAP | 0.013 |  | MAP | -0.022 | * | MAP | -0.023 |  | MAP | -0.033 | . |

Table S4: Results of linear mixed effect models for changes in CVs of the fifteen target species in response to mean annual precipitation (MAP). Given are results (Rm², Rc², test statistics and estimates for intercept and MAP) of the minimal adequate model (p < 0.1: .; p < 0.05: *; p < 0.01: **; p < 0.001: ***). Rm² = marginal R²; Rc² = conditional R².

|  | *Agropyron cristatum* | | | *Allium polyrrhizum* | | | *Artemisia adamsii* | | | *Artemisia frigida* | | | *Chenopodium album* | | |  |
| --- | --- | --- | --- | --- | --- | --- | --- | --- | --- | --- | --- | --- | --- | --- | --- | --- |
|  | Rm² = | 0.01 |  | Rm² = | 0.01 |  | Rm² = | 0.07 |  | Rm² = | 0.04 |  | Rm² = | 0.02 |  | |
|  | Rc² = | 0.41 |  | Rc² = | 0.24 |  | Rc² = | 0.37 |  | Rc² = | 0.33 |  | Rc² = | 0.47 |  | |
|  | Chi² = | 0.12 |  | Chi² = | 0.06 |  | Chi² = | 0.01 |  | Chi² = | 0.01 |  | Chi² = | 0.01 |  | |
| Estimate | Intercept | 0.264 | *** | Intercept | 0.259 | ** | Intercept | 0.183 | *** | Intercept | 0.290 | ** | Intercept | 0.269 | ** | |
|  | MAP | -0.005 |  | MAP | 0.006 |  | MAP | 0.054 |  | MAP | -0.043 | * | MAP | -0.040 |  | |
|  |  |  |  |  |  |  |  |  |  |  |  |  |  |  |  | |
|  | Cleistogenes squarrosa | | | Convolvulus ammanii | | | Dontostemon integrifolius | | | Elymus chinensis | | | Heteropappus altaicus | | |  |
|  | Rm² = | 0.01 |  | Rm² = | 0.02 |  | Rm² = | 0.29 |  | Rm² = | 0.01 |  | Rm² = | 0.04 |  | |
|  | Rc² = | 0.75 |  | Rc² = | 0.51 |  | Rc² = | 0.35 |  | Rc² = | 0.48 |  | Rc² = | 0.48 |  | |
|  | Chi² = | 0.01 |  | Chi² = | 0.01 |  | Chi² = | 0.58 |  | Chi² = | 0.01 |  | Chi² = | 0.01 |  | |
| Estimate | Intercept | 0.365 | ** | Intercept | 0.195 | ** | Intercept | 0.581 | *** | Intercept | 0.248 | *** | Intercept | 0.306 | ** | |
|  | MAP | -0.041 |  | MAP | -0.024 | . | MAP | -0.317 |  | MAP | 0.004 |  | MAP | -0.060 | * | |
|  |  |  |  |  |  |  |  |  |  |  |  |  |  |  |  | |
|  | *Koeleria cristata* | | | *Potentilla bifurca* | | | *Ptilotrichum canescens* | | | *Stipa glareosa* | | | *Stipa krylovii* | | |  |
|  | Rm² = | 0.04 |  | Rm² = | 0.08 |  | Rm² = | 0.01 |  | Rm² = | 0.07 |  | Rm² = | 0.01 |  | |
|  | Rc² = | 0.36 |  | Rc² = | 0.61 |  | Rc² = | 0.51 |  | Rc² = | 0.33 |  | Rc² = | 0.62 |  | |
|  | Chi² = | 0.07 |  | Chi² = | 0.01 |  | Chi² = | 0.01 |  | Chi² = | 0.05 |  | Chi² = | 0.01 |  | |
| Estimate | Intercept | 0.382 | *** | Intercept | 0.164 | * | Intercept | 0.240 | ** | Intercept | 0.119 | *** | Intercept | 0.307 | * | |
|  | MAP | -0.073 |  | MAP | 0.061 | ** | MAP | 0.002 |  | MAP | -0.135 |  | MAP | -0.027 |  | |
